# The Hippo-YAP signaling pathway promotes hepatocellular carcinoma progression by inducing FHL3 expression

**DOI:** 10.1038/s41419-025-08117-7

**Published:** 2025-11-03

**Authors:** Dean Rao, Tiantian Wang, Chengpeng Yu, Hanhua Dong, Wei Yan, Chenan Fu, Yiming Luo, Junli Lu, Zhoubing Sun, Huifang Liang, Wenjie Huang, Limin Xia

**Affiliations:** 1 Hepatic Surgery Centre, Tongji Hospital, Tongji Medical College, Huazhong University of Science and Technology, Hubei Key Laboratory of Hepato-Pancreato-Biliary Diseases, Wuhan, Hubei China; 2 Clinical Medicine Research Center for Hepatic Surgery of Hubei Province; Key Laboratory of Organ Transplantation, Ministry of Education and Ministry of Public Health, Wuhan, Hubei China; 3 State Key Laboratory of Holistic Integrative Management of Gastrointestinal Cancers and National Clinical Research Center for Digestive Diseases, Xijing Hospital of Digestive Diseases, Fourth Military Medical University, Xi’an, China; 4 Department of Gastroenterology, Institute of Liver and Gastrointestinal Diseases, Hubei Key Laboratory of Hepato-Pancreato-Biliary Diseases, Tongji Hospital of Tongji Medical College, Huazhong University of Science and Technology, Wuhan, Hubei China

**Keywords:** Oncogenes, Tumour biomarkers

## Abstract

The Hippo pathway plays an important role in cell proliferation, differentiation, and cancer occurrence. Yes-associated protein 1 (YAP) is a key effector molecule of Hippo pathway. Previous studies have found abnormal YAP overexpression in many solid tumors, including hepatocellular carcinoma (HCC). Here, we attempt to explore the cancer-promoting mechanism of YAP in HCC. The target gene of Hippo-YAP pathway, Four and a half LIM domain protein 3 (FHL3), was screened by spontaneous hydrodynamic tumor model with YAP participation and two publicly HCC microarray sets. Western blot (WB) and immunohistochemical (IHC) showed high protein levels of FHL3 in tumor tissues and the expression of FHL3 was associated with poorer prognosis. The biological effect experiments showed that FHL3 significantly promoted the progression of HCC. FHL3 interacted with MYC-associated zinc finger protein (MAZ) to recruit MAZ binding to the G-quadruplexes (G4s) structure, which promoted Kirsten rat sarcoma viral oncogene homologue (KRAS) transcription and activation of its downstream signal. Down-regulating KRAS expression inhibited the promoting effect of YAP-FHL3 signaling on HCC. In addition, transactivation of FHL3 mediated by YAP was verified by luciferase reporter assay and chromatin immunoprecipitation (ChIP). FHL3 knockdown inhibited the tumor-promoting effect of YAP and significantly delayed the tumorigenesis and progression caused by YAP. Finally, clinical data validated the correlation between YAP, FHL3, and KRAS expression. In conclusion, we identified a new target of Hippo-YAP signaling, FHL3, which interacts with MAZ to promote KRAS transcription and downstream oncogenic signaling pathway activation, thereby promoting HCC progression.

## Introduction

There were 19.3 million new cases of cancer worldwide in 2020. Liver cancer is the sixth most common cancer, accounting for 4.7%, but contributes to 8.3% of deaths, making it the third most common cause of cancer-related death. [1] The main form of liver cancer is hepatocellular carcinoma (HCC), which accounts for approximately 90% of all cancer cases. [2, 3] The pathophysiology of HCC is a complex multistep process. The genetic predispositions of HCC patients, interactions between viral and nonviral risk factors, cellular microenvironment, involvement of immune cells, and chronic diseases such as fatty liver and cirrhosis contribute to the early origination and malignant transformation of HCC. [4] Abnormal activation of oncogenes and inactivation of tumor suppressor genes, resulting in dysregulation of cancer-related molecular signaling pathways, are important causes of genetic susceptibility to HCC. [4, 5] Therefore, understanding the genetic and molecular lesions that contribute to cancer progression is necessary to provide fundamental insights for clinical diagnosis and treatment.

The Hippo pathway was first identified as an evolutionarily conserved signaling pathway in genetic screening of Drosophila tissue. Subsequent assays revealed that the Hippo pathway plays an important role in controlling cell proliferation, apoptosis, and regulating tissue homeostasis. [6, 7] Mechanically, when the Hippo pathway is activated, the complex composed of mammalian sterile 20-related 1 and 2 kinases (MST1/2) and salvador 1 (SAV1) phosphorylates and activates the large tumor suppressor 1 and 2 kinases (LATS1/2) complex, which further phosphorylates the transcriptional coactivator Yes-associated protein 1/Transcriptional coactivator with PDZ-binding motif (YAP/TAZ), the core effector molecules of the Hippo pathway. Phosphorylated YAP/TAZ is degraded by 14-3-3 proteins or sequestered in the cytoplasm. When the Hippo pathway is silenced, dephosphorylated YAP/TAZ is transported to nucleus, where it binds to the TEA domain family member (TEAD) transcription factors (TEAD1-4) and mediates the expression of target genes. [8–10] There is ample evidence that Hippo signaling is one of the most common dysregulated signaling pathways in human cancer. [11, 12] Furthermore, approximately 30% of HCC patients exhibit dephosphorylation of tumor suppressors MST1/2 and inactivation of the kinase cascade of the Hippo pathway, followed by high YAP/TAZ activity, indicating the important role of the Hippo pathway and its target genes in HCC. [13] Therefore, further exploration of the mechanism by which the Hippo-YAP pathway regulates the occurrence and development of HCC will provide new entry points and intervention strategies for the effective treatment of HCC.

Four and a half LIM domain protein 3 (FHL3) belongs to the FHL LIM-protein family and consists of four complete LIM domains and a half LIM domain, which are connected by a number of amino acid residues. [14] Physiologically, FHL3 is expressed in muscle tissue, affects muscle metabolism, and regulates gene expression and myoblast differentiation. [15] Studies have shown that FHL3 is expressed mainly in the nucleus and interacts with transcription factors such as phosphorylated cAMP response element binding protein (pCREB), myogenic determination gene number 1 (MyoD), myeloid zinc finger 1 (MZF-1) to play a transcriptional regulatory role and affect the expression of downstream functional genes. [16, 17] There is increasing evidence that FHL3 is also abnormally expressed in tumor tissues, affecting the progression and prognosis of various cancers such as gastric cancer, pancreatic cancer, and colorectal cancer. [18–20] However, there are few reports about FHL3 in HCC, and the expression and function of FHL3 in HCC still lack extensive studies.

In our study, we generated a spontaneous HCC model with abnormal YAP expression via hydrodynamic injection. [21] Transcriptome sequencing revealed that FHL3 may be the most upregulated gene associated with YAP in spontaneous HCC mouse models. Subsequent studies revealed that FHL3 is upregulated in HCC tissues and associated with poor prognosis in HCC patients, and that FHL3 can promote the proliferation and invasion of HCC cells both in vitro and in vivo. Furthermore, FHL3 recruits the transcription factor MYC-associated zinc finger protein (MAZ) to the G4s structure of the Kirsten rat sarcoma viral oncogene homolog (KRAS) promoter, which promotes the transcriptional activation of KRAS. In conclusion, we focused on the potential target genes of the Hippo-YAP pathway, its influence on the occurrence and development of HCC, and the mechanisms involved, suggesting that FHL3 may be a key target for screening or treatment of HCC patients with Hippo-YAP pathway abnormalities

## Materials and Methods

### Animal models

A xenograft assay was used to determine the growth ability of the tumor cells in vivo. To perform the xenograft tumorigenicity assay, 2 × 10^6^ tumor cells suspended in 100 μl of serum-free DMEM and Matrigel were injected subcutaneously into BALB/c nude mice at 5 weeks of age. Tumor progression was observed and measured every 3 days, and the mice were killed with carbon dioxide when the largest subcutaneous tumor approached but did not exceed 1.5 cm. The subcutaneous tumors were removed, weighed, measured, photographed, and fixed in 4% paraformaldehyde. Sections were taken for H&E and Ki67 staining.

For the intrahepatic orthotopic tumor xenotransplantation experiments, 1 × 10^6^ tumor cells were suspended in 30 μl of serum-free DMEM supplemented with Matrigel and injected into the livers of the nude mice. The mice were anesthetized and kept warm by heating pads. After surgery, the mice were nursed until they awoke from anesthesia. After 6 weeks, the mice were anesthetized again, and bioluminescent images were obtained via the Spectral Instruments Imaging Lago X system. The mice were killed with carbon dioxide after imaging, and the tumor tissue was measured and weighed. Livers removed from mice were fixed with 4% paraformaldehyde. The lungs removed from mice were irrigated with PBS and then injected with 4% paraformaldehyde and ligated.

The HTVi HCC model was constructed in 5-6-week-old C57BL/6 J mice. SB (7.5 μg), pT3-c-MYC (22.5 μg), pT3-AKT (22.5 μg) and pT3-YAP (22.5 μg) or pT3-Vector (22.5 μg) were co-dissolved in 2 ml of normal saline. The mixed plasmid mixture was injected into the mice through the tail vein within 5–10 s. For the AAV-8 adenovirus infection model, purified adenovirus was injected into mice through the tail vein.

All animal experiments were approved by the Ethics Committee of Tongji Hospital Affiliated to Huazhong University of Science and Technology and conducted in accordance with institutional guidelines for the care and use of laboratory animals.

### Statistical analysis

Differential expression analysis of cancer and paracancerous tissues was performed via *t*-tests. The relationships between FHL3 expression and various clinical features were analyzed via the chi-square test or Fisher’s exact test. Survival analysis was performed via the survminer R package. The prognosis was analyzed by the log-rank test, univariate Cox regression, and multivariate Cox regression. The means of the groups were analyzed via one-way ANOVA or two-way ANOVA. The bioinformatics analysis and statistical analysis were performed in R version 4.2.3. Graphpad Prism 8.0 was used for visualization.

Please see the Supplementary Information for detailed materials and methods.

## Results

### FHL3 is an abnormally expressed gene induced by YAP in HCC

**Fig. 1:**
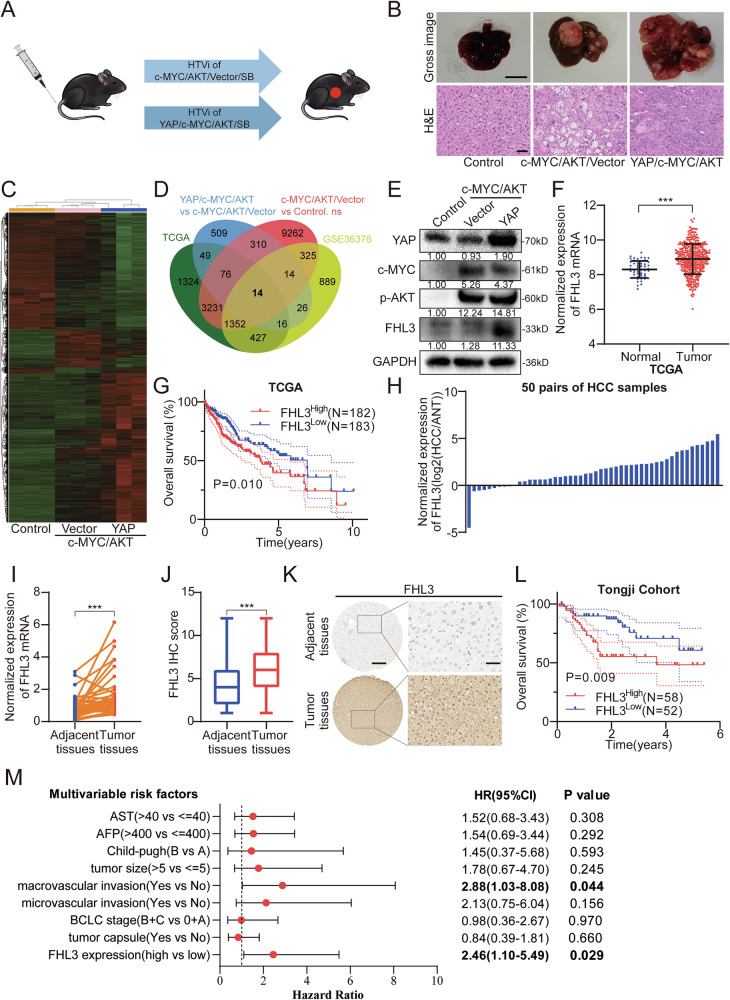
FHL3 is highly expressed in hepatocellular carcinoma and is associated with poor prognosis. **A** The mouse spontaneous tumor model driven with tumor driver genes. **B** Representative liver images (top) Scale bar: 1 cm, and H&E staining images (bottom) Scale bar: 50 μm, of the mouse spontaneous tumor model. **C** The heat map showing transcriptomic differences between the three groups of model tissues. **D** Four gene sets were used to screen the differential genes most associated with YAP, including the List 1: The gene set with Pearson correlation with YAP greater than 0.3 in the HCC expression matrix downloaded from TCGA. List 2: The gene set with significant differences between c-MYC/AKT/YAP group and c-MYC/AKT group (LogFC > 2). List 3: The gene set with no significant differences between c-MYC/AKT group and control group (LogFC < 2 and LogFC > -2). List 4: The gene set with Pearson correlation with YAP greater than 0.3 in the GSE36376 expression matrix downloaded from GEO database. **E** The expressions of YAP, c-MYC, p-AKT and FHL3 in liver tissue of animal model were detected by WB assay. **F** The expression level of FHL3 in cancer tissues and adjacent tissues in LIHC expression matrix of TCGA database. **G** The Kaplan-Meier plots of the OS rates of groups with FHL3 differential expression in LIHC expression matrix of TCGA database. **H** The protein differential expression of FHL3 in 50 pairs of cancerous tissues and adjacent tissues. **I** qPCR showed the expression of FHL3 mRNA in 50 pairs of cancer and adjacent tissues. **J** The expression of FHL3 in cancer tissues and adjacent tissues measured by immunohistochemical staining. **K** Representative images of FHL3 expression levels detected by immunohistochemical staining. Scale bar: overview images, 50 μm; magnified images, 200 μm. **L** The Kaplan–Meier plots of the OS rates of groups with FHL3 differential expression in Tongji cohort. **M** The Forest plot of the multivariate Cox proportional hazards model for overall survival of FHL3. Data are represented as mean ± SEM. ns not significant, *P < 0.05, **P < 0.01, ***P < 0.001.

The hydrodynamic transfection model enables long-term expression of target genes in mouse hepatocytes through hydrodynamic gene delivery combined with somatic cell integration mediated by Sleeping Beauty (SB), which is crucial for verifying the carcinogenic potential of abnormally altered carcinogenic signaling pathways. [22] To investigate the genetic landscape of the liver following Hippo pathway abnormalities, we first constructed a hydrodynamically transfected spontaneous HCC model driven by abnormal expression of c-MYC/AKT/vector or c-MYC/AKT/YAP via hydrodynamic transduction (Figs.1A, B and S1A, B). [21] RNA-seq revealed changes in genomic expression trends during tumorigenesis and tumor progression (Fig. 1C). To identify the most critical target genes of the Hippo-YAP pathway in the tumorigenic process of hepatocellular carcinoma, we constructed 4 genomes and obtained 14 genes from their intersection(Fig. 1D and Supplementary Table S3), which included reported YAP-regulated genes such as melanoma cell adhesion molecule (MCAM), SRY-box transcription factor 9 (SOX9), and connective tissue growth factor (CTGF). [23–25] To identify the key target genes involved in the tumorigenesis process, we eliminated genes related to differential expression in tumor tissue and adjacent normal tissue and the prognosis of these 14 genes in The Cancer Genome Atlas Program (TCGA), [26] and ultimately determined via RT-qPCR that FHL3 may be the gene most related to YAP (Fig. S1C, D). WB and immunohistochemistry (IHC) confirmed the significantly increased expression of FHL3 in the YAP/c-MYC/AKT group (Figs.1E and S1A, B).

### FHL3 is highly expressed in HCC and is associated with poor prognosis

To determine whether FHL3 is dysregulated in HCC, we first analyzed the expression level of FHL3 in the HCC patient cohort in the TCGA database and Gene Expression Omnibus (GEO) database (GSE36376). [27] Bioinformatics analysis revealed that the mRNA level of FHL3 in tumor tissues was significantly greater than that in adjacent normal tissues (Figs.1F and S1E). In addition, in the TCGA patient cohort, patients in the high FHL3 expression group had worse overall survival (OS) (Fig. 1G). In 50 pairs of mRNA samples and 50 pairs of protein samples from the Tongji cohort, RT-qPCR and WB results revealed that the expression of FHL3 in tumor tissues was significantly greater than that in adjacent normal tissues (Figs.1H, I and S1F, G). Furthermore, we detected FHL3 expression in 110 pairs of paraffin-embedded tissue samples from the Tongji cohort via IHC staining. The results revealed that FHL3 was expressed mainly in the nucleus, which was consistent with previous results [14, 28], and the expression of FHL3 in tumor tissues was greater than that in adjacent tissues (Fig. 1J, K). Moreover, we found that patients with high FHL3 expression had worse OS and disease-free survival (DFS) (Figs.1L and S1H). Chi-square test revealed that high FHL3 expression was associated with clinical features such as larger tumor volume, vascular invasion, and higher tumor grade (Supplementary Table S4). Univariate Cox regression analysis and multivariate Cox regression analysis also revealed that high FHL3 expression was an independent risk factor for poor prognosis (Figs.1M and S1I).

### FHL3 promotes the proliferation, invasion, and migration of HCC cells in vitro

**Fig. 2:**
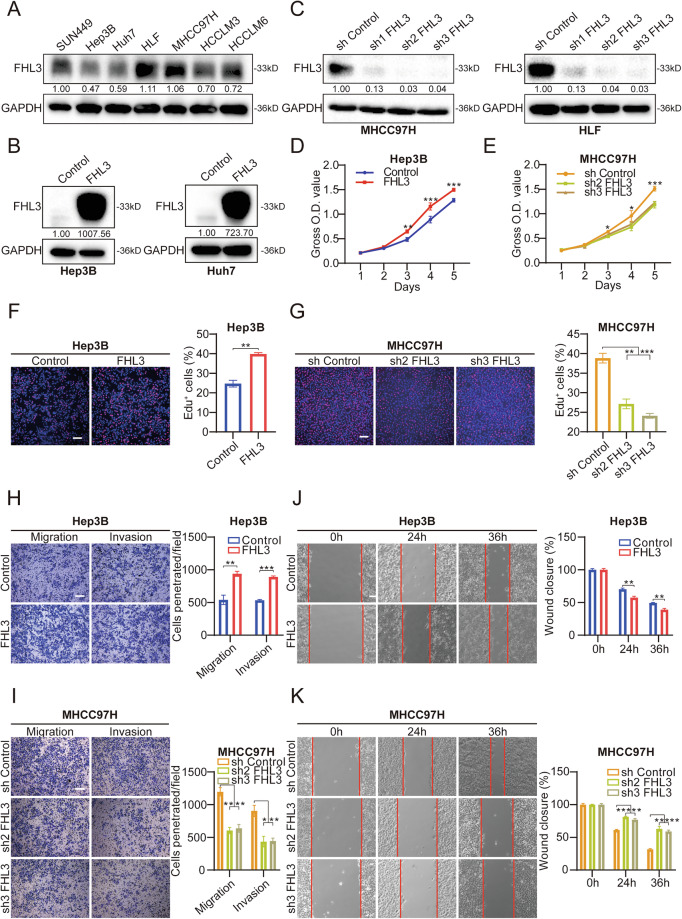
FHL3 promotes the proliferation, invasion, and migration of HCC cells in vitro. **A** FHL3 protein expression levels in several common HCC cell lines. **B** FHL3 stable overexpressing cell lines were constructed in Hep3B (left) and Huh7 (right). **C** FHL3 stable knock-down cell lines were constructed in MHCC97H (left) and HLF (right). **D** CCK8 assay in Hep3B cell line with stable overexpression of FHL3. Each experiment was repeated three times. **E** CCK8 assay in MHCC97H cell line with stable knockdown of FHL3. **F** Representative images of EdU incorporation assay in Hep3B cell line with stable overexpression of FHL3. Scale bar:100 μm. Each experiment was repeated three times. **G** Representative images of EdU incorporation assay in MHCC97H cell line with stable knockdown of FHL3. Scale bar:100 μm. **H** Representative images of Transwell assay in Hep3B cell line with stable overexpression of FHL3 and corresponding statistical graph. Scale bar: 100 μm. Each experiment was repeated three times. **I** Representative images of Transwell assay in MHCC97H cell line with stable knockdown of FHL3 and corresponding statistical graph. Scale bar: 100 μm. **J** Representative images of Scratch assay in Hep3B cell line with stable overexpression of FHL3 and corresponding statistical graph. Scale bar: 100 μm. Each experiment was repeated three times. **K** Representative images of Scratch assay in MHCC97H cell line with stable knockdown of FHL3 and corresponding statistical graph. Scale bar: 100 μm. Data are represented as mean ± SEM. ns not significant, *P < 0.05, **P < 0.01, ***P < 0.001.

To investigate the potential biological function of FHL3 in tumor cells, we first detected FHL3 expression levels in seven HCC cell lines (Fig. 2A). In accordance with the differences in the endogenous expression of FHL3 in HCC cell lines, Hep3B and Huh7 cell lines with low endogenous expression of FHL3 were selected to construct FHL3 overexpressing cell lines, and MHCC97H and HLF cell lines with high endogenous expression of FHL3 were constructed to construct FHL3-stable knockdown cell lines (Fig. 2B, C). To investigate the effect of FHL3 on the proliferation ability of HCC cell lines, we conducted CCK-8 assays and EdU incorporation assays. The results revealed that FHL3 overexpression significantly increased the proliferation of Hep3B and Huh7 cells, whereas FHL3 knockdown inhibited the proliferation of MHCC97H and HLF cell lines (Figs.2D–G and S2A–D). In addition, we explored the effects of abnormal FHL3 expression on the invasion and migration ability of HCC cell lines via transwell and scratch assays. The results revealed that FHL3 overexpression promoted the invasion and migration of the Hep3B and Huh7 cell lines, whereas FHL3 knockdown had the opposite effect on the MHCC97H and HLF cell lines (Figs.2H–K and S2E–H). In general, FHL3 promotes the proliferation, invasion, and metastasis of HCC cell lines in vitro.

### FHL3 promotes tumor growth and lung metastasis in vivo

**Fig. 3:**
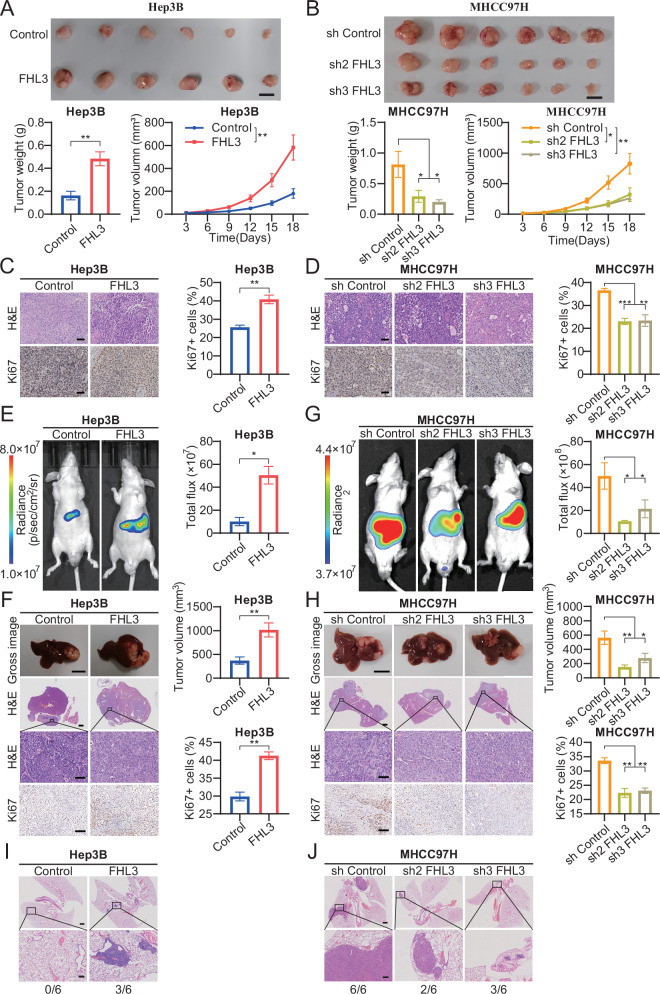
FHL3 promotes tumor growth and lung metastasis in vivo. **A** Subcutaneous tumor implantation models in Hep3B cell lines with stable overexpression of FHL3 (top), and tumor weight and volume statistics (bottom). *n* = 6. Scale bar: 1 cm. **B** Subcutaneous tumor implantation models in MHCC97H cell lines with stable knockdown of FHL3 (top), and tumor weight and volume statistics (bottom). *n* = 6. Scale bar: 1 cm. **C** Representative images of H&E staining and immunohistochemical staining of subcutaneous tumor tissue implanted with Hep3B cell line (left), and statistics of Ki67 positive rate of cells (right). Scale bar: 100 μm. **D** Representative images of H&E staining and immunohistochemical staining of subcutaneous tumor tissue implanted with MHCC97H cell line (left), and statistics of Ki67 positive rate of cells (right). Scale bar: 100 μm. **E** Representative images of mouse imaging in situ implantation model of Hep3B cell line (left), and flux statistics (right). *n* = 6. **F** Representative images of Liver (Scale bar: 1 cm), H&E staining of overview images (Scale bar: 1 mm), magnified images (Scale bar: 100 μm) and Ki67 immunohistochemical staining (Scale bar: 100 μm, left), and statistics of tumor volume and proportion of Ki67 (right) in situ implantation model of Hep3B cell line. **G** Representative images of mouse imaging in situ implantation model of MHCC97H cell line (left), and flux statistics (right). *n* = 6. **H** Representative images of Liver (Scale bar: 1 cm), H&E staining of overview images (Scale bar: 1 mm), magnified images (Scale bar: 100 μm) and Ki67 immunohistochemical staining (Scale bar: 100 μm, left), and statistics of tumor volume and proportion of Ki67 (right) in situ implantation model of MHCC97H cell line. **I** Representative images of lung H&E staining and lung metastasis statistics from in situ implantation models of Hep3B cell lines. Scale bar: overview images, 1 mm; magnified images, 200 μm. **J** Representative images of lung H&E staining and lung metastasis statistics from in situ implantation models of MHCC97H cell lines. Scale bar: overview images, 1 mm; magnified images, 200 μm. Data are represented as mean ± SEM. ns: not significant, *P < 0.05, **P < 0.01, ***P < 0.001.

Previous results have demonstrated that FHL3 promotes the proliferation, invasion, and migration of HCC cell lines in vitro. Therefore, we further explored the effects of FHL3 on tumor growth and lung metastasis in vivo. In a subcutaneous xenotransplantation assay in nude mice, we found that the volume and weight of tumors in the Hep3B group with stable FHL3 overexpression were significantly greater than those in the control group (Fig. 3A). In contrast, FHL3 knockdown inhibited the increase in tumor volume and weight in the MHCC97H group (Fig. 3B). Ki67 IHC staining of mouse subcutaneous tumors revealed more Ki67-positive staining in the FHL3 overexpressing group, and the opposite trend was observed in FHL3 knockdown group (Fig. 3C, D). Moreover, the fluorescence intensity, tumor volume, and Ki67-positive rate were greater in the FHL3 overexpressing group than in the control group, whereas FHL3 knockdown hindered tumor growth (Fig. 3E–H). In addition, H&E staining of lung tissue revealed that the group with higher FHL3 expression levels had more lung metastases and more lung tumor nodules (Fig. 3I, J). These results suggest that FHL3 promotes tumor growth and lung metastasis in vivo.

### FHL3 interacts with MAZ

**Fig. 4:**
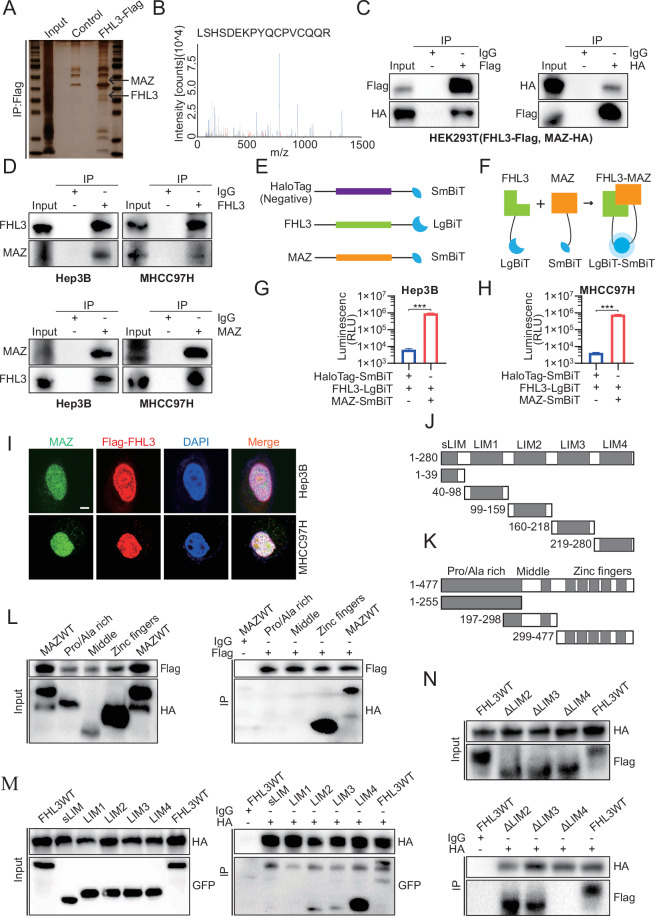
FHL3 interacts with MAZ. **A** The quality of mass spectrum samples is verified by silver staining. **B** The specific peptide of MAZ was detected by mass spectrometry. **C** The exogenous interaction between FHL3-Flag and MAZ-HA was verified in HEK293T cell line. **D** Endogenous interactions of FHL3 with MAZ were verified in Hep3B (left) and MHCC97H (right) cell lines. **E** Schematic diagram of FHL3-LargeBiT and MAZ-SmallBiT structures, with HaloTag-SmBiT used as a negative control. **F** Schematic illustration of FHL3 and MAZ interacting to make LargeBiT and SmallBiT combine to emit light. **G** NanoBiT Protein-Protein Interaction experiments show that the interaction of FHL3-LargeBiT and MAZ-SmallBiT in Hep3B releases bright signals of NanoLuc luciferase. **H** NanoBiT Protein – Protein Interaction experiments show that the interaction of FHL3-LargeBiT and MAZ-SmallBiT in MHCC97H releases bright signals of NanoLuc luciferase. **I** Immunofluorescence experiments showed that FHL3 was co-localized with MAZ. Scale bar: 5 μm. **J** Truncated schematic of FHL3. **K** Truncated schematic of MAZ. **L** IP showed that FHL3 interacts with the Zinc fingers (299–477) region of MAZ. **M** IP showed that MAZ interacts with LIM2 (99–159), LIM3 (160–218), and LIM4 (219–280) regions of FHL3. **N** IP showed that the absence of LIM4 (219–280) domain of FHL3 significantly affected its binding to MAZ.

Protein-protein interactions constitute a major component of cellular biochemical reaction networks, and protein-protein interaction networks and transcriptional regulatory networks are important for regulating cellular and biological functions. [29, 30] Previous studies have shown that the physiological function of FHL3 involves interactions with Yin Yang-1 (YY1), MyoD, hypoxia-inducible factor 1 (HIF-1), and other transcription factors to play a transcriptional synergy or repression role, thereby regulating tissue differentiation, angiogenesis, metabolic reprogramming, and other physiological functions. [15, 16, 31] We enriched the protein interacting with the FHL3 protein via co-IP and confirmed the high abundance of the FHL3 protein via silver staining (Fig. 4A). A total of 84 proteins with specificity and potential binding to FHL3 were identified by MS in the FHL3-overexpressing group. Considering the potential functional properties of FHL3 as a transcriptional cofactor and its predominant nuclear localization, we selected the top 10 nuclear-localized proteins from the candidate proteins (Fig. S3A). Notably, MAZ represents a classical DNA-binding transcription factor among these candidates (Fig. 4B). MAZ has been reported to be upregulated in HCC and to promote HCC progression by activating the transcription of the oncogenes CCND1, NEIL3, etc. [32, 33] In addition, the interaction between the FHL3 and MAZ proteins was validated by exogenous IP in HEK293T cells and endogenous IP in Hep3B and MHCC97H cells (Fig. 4C, D). To further investigate the interaction between FHL3 and MAZ, we conducted a NanoBiT protein-protein interaction assay. [34] The LargeBiT and SmallBiT were fused with the C-terminal of FHL3 and MAZ, respectively. HaloTag was used as a negative control (Fig. 4E, F). The cells with FHL3-LargeBiT and MAZ-SmallBiT expression showed significantly higher chemiluminescence activity than those of cells with FHL3-LargeBiT and HaloTag-SmallBiT expression (Fig. 4G, H). In addition, on the basis of the immunofluorescence verification of the suitability of antibodies to FHL3 and MAZ, an immunofluorescence assay confirmed the colocalization of the FHL3 protein and MAZ protein in the nucleus (Fig. 4I and S3B–E). However, despite the presence of mutual binding, FHL3 did not affect the protein abundance of MAZ (Fig. S3F, G). To further verify the binding sites of FHL3 and MAZ interactions, we truncated proteins according to the domains of FHL3 and MAZ and fused GFP tags on the C-terminus of each domain of FHL3 and HA tags on the C-terminus of each domain of MAZ (Fig. 4J, K). The results revealed that FHL3 interacts with the zinc finger domain of MAZ (Fig. 4L). Interestingly, MAZ interacted with the LIM4 (219-280) domain, and also weakly bound to the LIM2 (99-159) and LIM3 (160-218) domains (Fig. 4M). Moreover, the deletion of the LIM4 domain eliminated the interaction between FHL3 and MAZ, whereas deletion of LIM2 or LIM3 alone had little effect on the interaction between FHL3 and MAZ (Fig. 4N). To verify whether the LIM4 domain of FHL3 has reproducible cancer-promoting functions, we transiently transfected LIM2, LIM3, and LIM4 deletion mutants into Hep3B cells (Fig. S4A). The results of the CCK-8, EdU, transwell, and scratch assays revealed that deletion of the LIM4 domain of FHL3 significantly reduced the cancer-promoting function of FHL3, whereas deletion of the LIM2 or LIM3 domain had little effect on the cancer-promoting function of FHL3 (Fig. S4B–E). These results suggest that the LIM4 domain of FHL3 is indispensable for the carcinogenic function of FHL3 because it interacts with MAZ.

### FHL3 regulates the transcriptional activity of MAZ by influencing the binding of MAZ to G4s

**Fig. 5:**
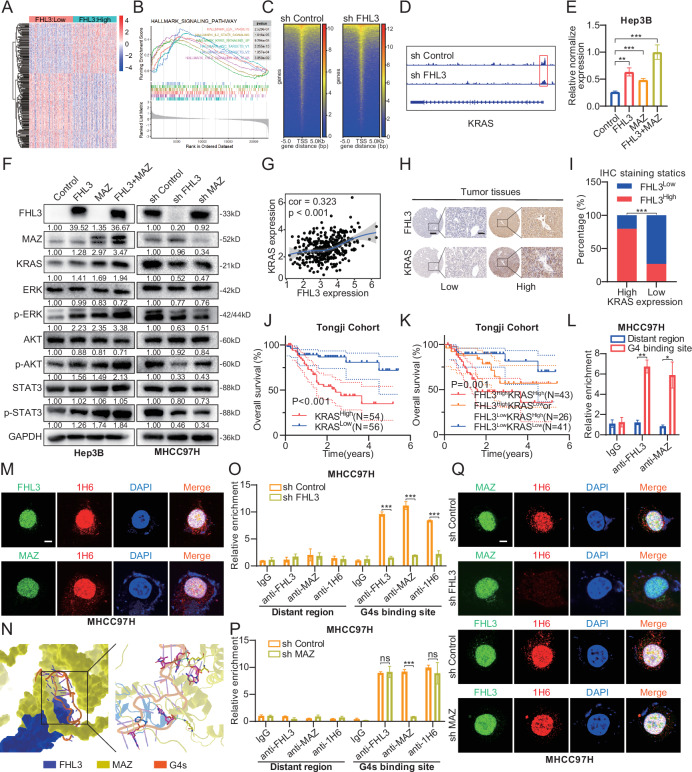
FHL3 regulates the transcriptional activity of MAZ by influencing the binding of MAZ to G4s. **A** Heat maps showed that there were many differentially expressed genes in FHL3 differential analysis based on LIHC expression matrix of TCGA database. **B** GSEA enrichment analysis of differential genes showed that multiple pathways were enriched. **C**, **D** Heat maps (left) show the effect of FHL3 knockdown on MAZ binding to the promoter region. Only in the presence of FHL3 does MAZ bind to the promoter region of KRAS (right bottom). **E** qPCR experiments showed the effect of single or simultaneous overexpression of FHL3 and MAZ on the transcription levels of KRAS. **F** WB experiment showed the effects of overexpression or knockdown of FHL3 and MAZ on KRAS protein level and its downstream signaling pathway. **G** The correlation between FHL3 and MAZ was calculated based on LIHC expression matrix in TGCA database. **H** Immunohistochemical staining showed the difference of FHL3 and KRAS protein expression in the same tissue. Scale bar: overview images, 50μm; magnified images, 200 μm. **I** Immunohistochemical staining scores of FHL3 and KRAS showed that the protein abundances of the two were correlated. **J** The Kaplan–Meier plots of the OS rates of groups with KRAS differential expression in Tongji cohort. **K** The Kaplan–Meier plots of the OS rates of groups with FHL3 and KRAS differential expression in Tongji cohort. **L** ChIP experiments showed that FHL3 and MAZ bind to the G4s DNA region of the KRAS promoter in wild-type MHCC97H cell line. **M** Immunofluorescence showed that FHL3 or MAZ proteins were co-located with the G4s DNA region of KRAS promoter in wild-type MHCC97H cell line. Scale bar: 5 μm. **N** Molecular docking experiments showed the interaction of FHL3, MAZ and G4s. **O** ChIP experiments showed that FHL3 knockdown in MHCC97H cell lines resulted in structural changes in the G4s DNA region of the KRAS promoter (the G4s DNA structure is not recognized by anti-1H6 antibodies), thus preventing MAZ from binding to this site. **P** ChIP experiments showed that MAZ knockdown in MHCC97H cell lines had no effect on the structure of the G4s DNA region of the KRAS promoter or its binding to FHL3. **Q** Immunofluorescence revealed loss of colocalization of the MAZ protein and the G4s DNA region of KRAS promoter in the Hep3B cell line with FHL3 knockdown (top). However, the co-localization of the FHL3 protein and the G4s DNA region of KRAS promoter was not affected in the case of MAZ knockdown (bottom). Scale bar: 5 μm. Data are represented as mean ± SEM. ns: not significant, *P < 0.05, **P < 0.01, ***P < 0.001.

To further explore the downstream target genes that FHL3 synergistically activates by recruiting MAZ, the HCC gene expression matrix in the TCGA database was divided into two groups according to the level of FHL3 expression (Fig. 5A). GSEA of the results revealed that the E2F target gene set, the IL2-STAT5 signaling pathway, the KRAS signaling pathway, the MYC target gene set and the TNF-α signaling pathway were enriched in the high FHL3 expression group (Fig. 5B). Moreover, we explored the effect of FHL3 on the binding of MAZ to the target gene promoter sequence via CUT&Tag analysis. The results revealed that MAZ could bind to the KRAS promoter region in the control group, but was significantly inhibited by FHL3 knockdown (Fig. 5C, D). The dysregulation of wild-type KRAS expression is also closely related to HCC progression, sensitivity, and resistance to chemotherapy drugs. [35, 36] We examined the regulatory role of FHL3 and MAZ on KRAS expression. RT-qPCR showed that overexpression of FHL3 or MAZ could upregulate the mRNA level of KRAS, and the simultaneous overexpression of FHL3 and MAZ had a more significant effect on the upregulation of KRAS. When FHL3 and MAZ were knocked down, the mRNA level of KRAS was also down-regulated (Figs.5E and S5A). Similar changes in KRAS protein levels were also observed in WB assay (Fig. 5F). Furthermore, the downstream signaling pathways of KRAS were also determined. It was found that the activity of the classical downstream signaling pathways ERK, PI3K-AKT, and STAT3 changes with the level of KRAS protein (Fig. 5F). In addition, correlation analysis of TCGA database indicated that both FHL3 and MAZ were correlated with KRAS mRNA levels in HCC patients (Figs.5G and S5B). Finally, we performed IHC staining of KRAS in 110 pairs of tissue sample array. The results showed that KRAS was also highly expressed in HCC tumor tissues (Fig. S5C, D). Moreover, we found that the expression of KRAS had a similar trend to that of FHL3 (Fig. 5H, I). In addition, patients with high KRAS expression tended to have poorer OS and DFS (Figs. 5J and S5E). Moreover, patients with high expression of both FHL3/KRAS had significantly poorer OS and DFS than patients with low expression of both FHL3/KRAS, and even worse than patients with high expression of one of the FHL3/KRAS (Figs.5K and S5F).

Next, we further investigated the mechanisms of FHL3-induced KRAS expression. Studies have shown that the promoter region of many oncogenes, including KRAS, has a G-rich 4-strand nucleotide structure, also known as G4s [37], and that MAZ can bind to this structure to activate KRAS transcription (Fig. S5G). [38, 39] To explore whether FHL3 and MAZ bind to the G4s structure of the KRAS promoter, a ChIP assay was performed. The results revealed that both the FHL3 and MAZ antibody groups bound more G4s DNA fragments than the IgG antibody group did (Figs.5L and S5H). In addition, a G4s structure-specific antibody, 1H6, has been widely used in studies to analyze the G4s structure in cells. [39–41] Immunofluorescence revealed that both FHL3 and MAZ colocalized with G4s in the nucleus (Figs.5M and S5I). To explore the spatial structure of FHL3 and MAZ binding to G4s, protein-DNA molecular docking experiments were performed. The results revealed that the LIM4 domain of FHL3 and the zinc fingers of MAZ interact with special G4s structures. Moreover, Phe263 of FHL3, which belongs to the LIM4 domain, is indispensable for its binding with G4s, revealing that LIM4 is necessary for the function of FHL3. Specifically, FHL3 decreased the interface binding free energy of MAZ/G4s. (Figs.5N and S5J and Supplementary Table S5–7). In addition, ChIP assays revealed that FHL3 knockdown decreased the binding of MAZ to G4s, whereas MAZ knockdown did not affect the binding of FHL3 to G4s (Figs.5O, P and S5K, L). Consistent with the ChIP results, the results of the immunofluorescence experiments revealed that FHL3 knockdown decreased the stability of G4s, thereby impairing MAZ-mediated transcriptional regulation (Figs.5Q and S5M). These results indicate that FHL3 stabilizes the G4s structure of the KRAS promoter and recruits MAZ to activate KRAS transcription.

### FHL3 promotes HCC progression through the KRAS signaling pathway

**Fig. 6:**
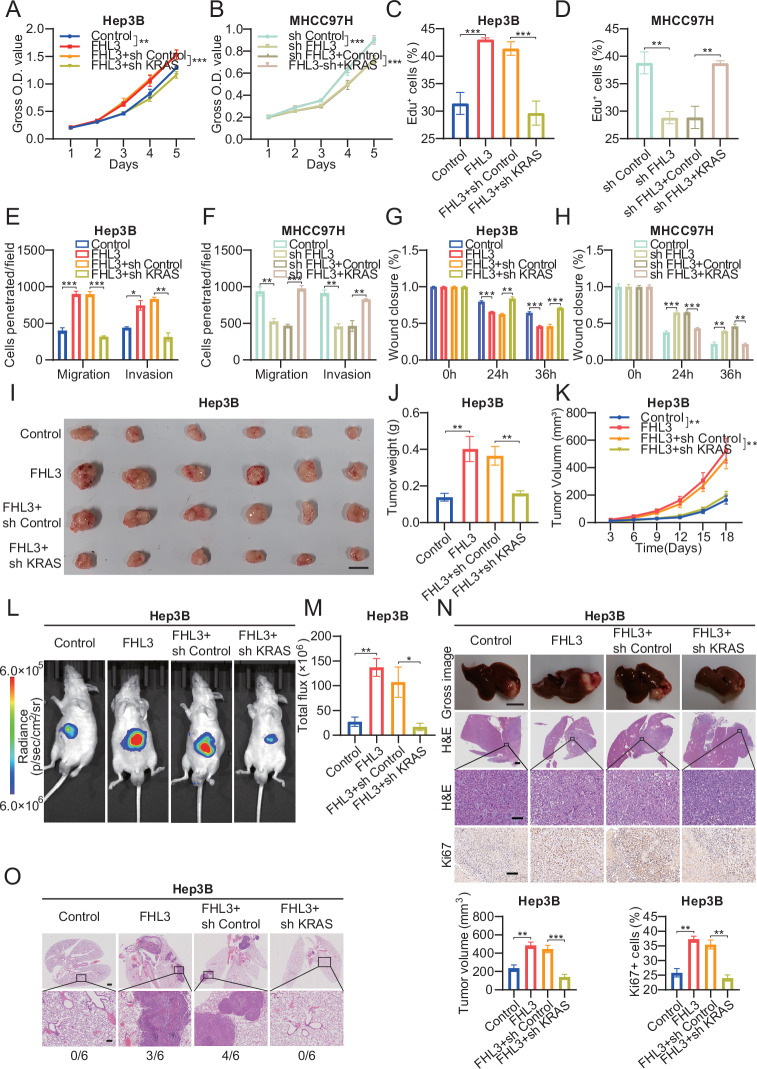
FHL3 promotes HCC progression through KRAS signaling pathway. CCK8 assays of KRAS knockdown on the basis of FHL3 overexpression in Hep3B cell line (**A**) or KRAS overexpression on the basis of FHL3 knockdown in MHCC97H cell line (**B**). Each experiment was repeated three times. EdU incorporation of KRAS knockdown on the basis of FHL3 overexpression in Hep3B cell line (**C**) or KRAS overexpression on the basis of FHL3 knockdown in MHCC97H cell line (**D**). Each experiment was repeated three times. Transwell assays of KRAS knockdown on the basis of FHL3 overexpression in Hep3B cell line (**E**) or KRAS overexpression on the basis of FHL3 knockdown in MHCC97H cell line (**F**). Each experiment was repeated three times. Scratch assays of KRAS knockdown on the basis of FHL3 overexpression in Hep3B cell line (**G**) or KRAS overexpression on the basis of FHL3 knockdown in MHCC97H cell line (**H**). Each experiment was repeated three times. **I** Subcutaneous tumor implantation experiment with KRAS knockdown based on overexpression of FHL3 in Hep3B cell line. *n* = 6. Scale bar: 1 cm. **J** Statistical map of subcutaneous tumor weight. **K** The volume of the subcutaneous tumor was measured every three days with the formula V = 1/2 L*H^2^. Representative images of mouse imaging in situ implantation model of KRAS knockdown on the basis of FHL3 overexpression in Hep3B cell line (**L**), and flux statistics (**M**). *n* = 6. **N** Representative images of Liver (Scale bar: 1 cm), H&E staining of overview images (Scale bar: 1 mm), magnified images (Scale bar: 100 μm), and Ki67 immunohistochemical staining (Scale bar: 100 μm, left), and statistics of tumor volume and proportion of Ki67 (right) in situ implantation model of Hep3B cell line. **O** Representative images of lung H&E staining and lung metastasis statistics from in situ implantation models of KRAS knockdown on the basis of FHL3 overexpression in Hep3B cell line. Scale bar: overview images, 1 mm; magnified images, 200 μm. Data are represented as mean ± SEM. ns: not significant, *P < 0.05, **P < 0.01, ***P < 0.001.

To verify whether FHL3 promotes HCC progression through the KRAS signaling pathway, we knocked down KRAS on the basis of FHL3 overexpression in the Hep3B cell line and overexpressed KRAS on the basis of FHL3 knockdown in the MHCC97H cell line. CCK-8 and EdU incorporation assays revealed that FHL3 overexpression promoted the proliferation of Hep3B cells. However, KRAS knockdown significantly weakened the effect of FHL3. Moreover, overexpression of KRAS also reversed the effect of FHL3 knockdown on cell proliferation (Figs.6A–D and S6A, B). Transwell and scratch assays revealed that FHL3 and KRAS had similar effects on the invasion and migration of HCC cell lines (Figs. 6E–H and S6C–F). In addition, KRAS knockdown inhibited FHL3-mediated promotion of subcutaneous tumor growth in mice, with a low percentage of Ki67-positive tumors (Figs.6I–K and S6G, H). In the liver orthotopic xenotransplantation assay, simultaneous knockdown of KRAS significantly reduced the stronger fluorescence intensity and larger tumor volume caused by FHL3 than did FHL3 overexpression alone (Fig. 6L–N). Moreover, knocking down KRAS decreased FHL3-induced lung metastasis (Fig. 6O). Overall, KRAS is a key target molecule that mediates FHL3 to promote HCC progression both in vivo and in vitro.

### FHL3 knockdown significantly inhibited the promoting effect of the Hippo-YAP signaling pathway on HCC

**Fig. 7:**
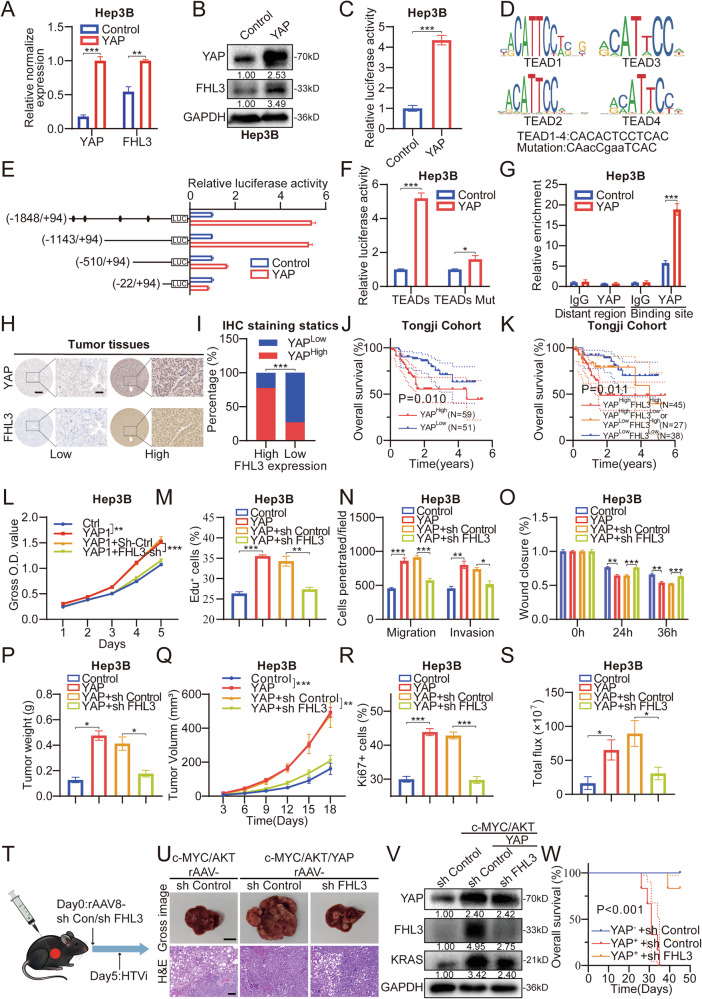
FHL3 knockdown significantly inhibited the promotion effect of Hippo-YAP signaling pathway on HCC. **A** qPCR showed that overexpression of YAP up-regulated FHL3 mRNA levels. **B** WB showed that overexpression of YAP up-regulated FHL3 protein levels. **C** Effects of overexpression of YAP on transcriptional activity of FHL3. **D** Potential DNA sequences bound to transcription factors TEAD1-4 (top) and mutated sequences (bottom). **E** Effect of YAP on transcriptional activity of FHL3 after truncating the promoter region of FHL3. **F** Effect of YAP on FHL3 transcriptional activity after mutation of TEAD1-4 most likely acting DNA sequence in the FHL3 promoter region. **G** ChIP assay verified the binding of YAP/TEAD to the FHL3 promoter region. **H** Immunohistochemical staining showed the difference of YAP and FHL3 protein expression in the same tissue. Scale bar: overview images, 50 μm; magnified images, 200 μm. **I** Immunohistochemical staining scores of YAP and FHL3 showed that the protein abundances of the two were correlated. **J** The Kaplan–Meier plots of the OS rates of groups with YAP differential expression in Tongji cohort. **K** The Kaplan–Meier plots of the OS rates of groups with YAP and FHL3 differential expression in Tongji cohort. **L** CCK8 assays of FHL3 knockdown on the basis of YAP overexpression in Hep3B cell line. Each experiment was repeated three times. **M** EdU incorporation of FHL3 knockdown on the basis of YAP overexpression in Hep3B cell line. Each experiment was repeated three times. **N** Transwell assays of FHL3 knockdown on the basis of YAP overexpression in Hep3B cell line. Each experiment was repeated three times. **O** Scratch assays of FHL3 knockdown on the basis of YAP overexpression in Hep3B cell line. Each experiment was repeated three times. **P** Statistical map of subcutaneous tumor weight. *n* = 6. **Q** The volume of the subcutaneous tumor was measured every three days. **R** Statistics of Ki67 positive rate of cells. **S** Total flux statistics of liver in situ implantation model. **T** The spontaneous tumor model driven by YAP was established after FHL3 knockdown by rAAV8 adenovirus method. n = 6. **U** Representative liver images (top) Scale bar: 1 cm, and H&E staining images (bottom) Scale bar: 50μm, after FHL3 knockdown in the YAP-driven mouse spontaneous tumor model. **V** The expressions of YAP, FHK3, KRAS in liver tissue of animal model were detected by WB assay. **W** Effect of FHL3 knockdown on the prognosis of YAP-induced HCC mice. Data are represented as mean ± SEM. ns: not significant, *P < 0.05, **P < 0.01, ***P < 0.001.

We further studied the molecular mechanism of FHL3 regulation by YAP. RT-qPCR and WB revealed that YAP upregulated FHL3 mRNA and protein levels in Hep3B cells (Fig. 7A, B). A luciferase reporter assay revealed that YAP significantly increased the transcriptional activity of the FHL3 promoter. (Fig. 7C). According to the binding sites of the classical transcription factor TEAD1-4 of the Hippo-YAP pathway predicted by JASPAR (https://jaspar.elixir.no/), we truncated the transcriptional regulatory sequence of FHL3. The results revealed that YAP regulated FHL3 transcription mainly through the −1143 to −510 segment, which contained only one TEAD1-4 common binding site (Fig. 7D, E). After mutation of this site, we observed a significantly reduced transcriptional activity of FHL3 (Fig. 7D, F). ChIP also demonstrated that YAP/TEAD binds to this regulatory sequence (Fig. 7G). In addition, transcriptional analysis of the LIHC dataset from TCGA revealed a correlation between the YAP and FHL3 mRNA levels (Fig. S7A). In the Tongji cohort, IHC revealed that the expression of YAP in cancer tissues was greater than that in adjacent tissues, and the expression of YAP was also significantly correlated with FHL3 expression. (Figs.7H, I and S7B, C). Furthermore, patients with high YAP expression had worse OS and DFS (Figs.7J and S7D). When FHL3 expression was included in the analysis, patients with high levels of both YAP and FHL3 expression had the worst prognosis (Figs.7K and S7E).

To further verify the indispensable role of FHL3 in the Hippo-YAP signaling pathway, we knocked down FHL3 via the overexpression of YAP in Hep3B cells. CCK-8 and EdU incorporation assays revealed that the overexpression of YAP promoted the proliferation of Hep3B cells. However, FHL3 knockdown significantly weakened the effect of YAP on Hep3B cells (Figs. 7L, M and S8A). Similarly, FHL3 knockdown antagonized the role of YAP in promoting the invasion and metastasis of Hep3B cell lines (Figs. 7N, O and S8B, C). Moreover, the results of the subcutaneous tumor model also suggested that FHL3 knockdown negated the proliferation promoting effect of YAP in mice (Figs. 7P–R and S8D, E). In addition, the orthotopic liver tumor assay results demonstrated that FHL3 knockdown reversed the increase in total flux, tumor volume, Ki67 positivity, and lung metastasis induced by YAP (Figs. 7S and S8F–J). Finally, the effect of blocking FHL3 on YAP induced HCC in mice with adenovirus-specific FHL3 knockdown. The results showed that FHL3 knockdown significantly hindered YAP/c-MYC/AKT-induced HCC progression. The liver morphology of the FHL3 knockdown group was more similar to that of the c-MYC/AKT group, with more fatty changes around the tumor observed by H&E, which is typical of c-MYC/AKT-induced early liver lesions (Figs.7T–W and S9). In addition, the expression of KRAS was decreased in the FHL3-knockdown group (Figs. 7V and S9). Taken together, these results suggest that FHL3 is essential for the oncogenic effects of the Hippo-YAP pathway. Blocking FHL3 resulted in a favorable prognosis for YAP-activated HCC mice.

### YAP and KRAS have similar expression characteristics and prognosis in HCC

**Fig. 8:**
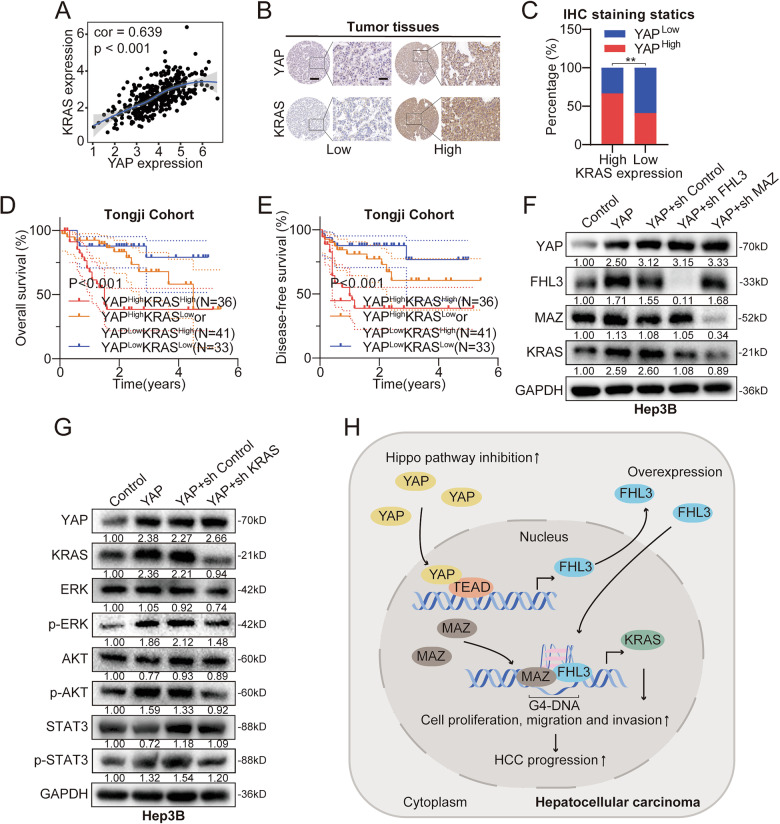
YAP and KRAS have similar expression characteristics in HCC and are associated with poor prognosis. **A** The correlation between YAP and KRAS was calculated based on LIHC expression matrix in TGCA database. **B** Immunohistochemical staining showed the difference of YAP and KRAS protein expression in the same tissue. Scale bar: overview images, 50 μm; magnified images, 200 μm. **C** Immunohistochemical staining scores of YAP and KRAS showed that the protein abundances of the two were correlated. **D** The Kaplan–Meier plots of the OS rates of groups with YAP and KRAS differential expression in Tongji cohort. **E** The Kaplan–Meier plots of the DFS rates of groups with YAP and KRAS differential expression in Tongji cohort. **F** WB data confirmed that YAP modulates KRAS expression in a manner dependent on FHL3 or MAZ. **G** WB experiment showed the effects of YAP and KRAS protein level on downstream signaling pathways. **H** Schematic model of the mechanism by which YAP promotes HCC progression through up-regulation of FHL3 expression.

Finally, we analyzed the correlation between YAP and KRAS expression patterns. TCGA online database analysis revealed a significant correlation between YAP and KRAS mRNA expression in HCC (Fig. 8A). IHC staining of tissue samples from the Tongji cohort revealed similar protein levels of YAP and KRAS; that is, HCC tissues with high YAP expression tended to have stronger KRAS staining levels (Fig. 8B, C). Moreover, patients with high expression of both YAP and KRAS had the worst OS and DFS (Fig. 8D, E). The WB results revealed that overexpression of YAP upregulates the protein level of KRAS, but this trend was reversed by knockdown of FHL3 or MAZ. Additionally, YAP overexpression activated the KRAS downstream signaling pathways to varying degrees, but this effect was reversed by knocking down KRAS (Fig. 8F). These results suggest that YAP and KRAS have similar expression patterns and prognosis, and that YAP may promote HCC progression by upregulating KRAS expression and the activity of its downstream oncogenic signaling pathway. Overall, YAP and KRAS have similar expression patterns and prognosis, and YAP may promote HCC progression by upregulating KRAS expression and the activity of its downstream oncogenic signaling pathway.

## Discussion

The Hippo pathway is an important tumor suppressor pathway, which is involved in regulating the growth and regeneration of liver tissue. [42, 43] Abnormal activation of YAP, a key gene in the Hippo pathway, is a major feature of many cancers, including HCC. [10] However, the molecular mechanism of YAP in the pathogenesis and development of HCC remains unclear. Therefore, we constructed a spontaneous mouse HCC model driven by the participation of YAP. Through bioinformatics analysis of public databases, we screened the FHL3 gene, which is associated with YAP expression and may play an important role in the formation of HCC in mice.

FHL3 belongs to the FHL protein family, with the special structure of LIM-only proteins. The LIM domain that makes up FHL3 is an enzymatically inactive protein-protein interaction domain that determines its function as a scaffold protein or adapter molecule. FHL3 reportedly recruits the transcription factors MyoD, CREB, and pCREB to regulate the composition of muscle fiber types during physiological processes. [14–16] The LIM3 domain has been reported to play an indispensable role in the interaction of FHL3 with the transcription factor GSK3β, suggesting that the four and a half domains of FHL3 do not have simple repetitive functions. [20] In addition, much evidence suggests that FHL3 is abnormally expressed in a variety of cancers and plays a dual role in influencing tumor proliferation and metastasis. [20, 28] In this study, we demonstrated that FHL3 is highly expressed in tumor tissues and is an independent risk factor that predicts poor clinical features and prognosis. Effect assays targeting FHL3 revealed that FHL3 can promote the proliferation, invasion, and metastasis of HCC.

MAZ is located on human chromosome 16p11.2, and the translated product, which is encoded by 477 amino acids, is widely expressed in the human heart, liver, and other tissues. [38] Previous studies have shown that MAZ is a transcription factor with six Cys2His2-type zinc finger moieties at the carboxyl terminus that play dual roles in the initiation and termination of gene transcription. [44] MAZ activates the transcription of several oncogenes, suggesting that the abnormal expression of MAZ is closely related to the occurrence and development of tumors. [32, 38] Transcriptional regulation of MAZ is based on the interaction between the GC-rich DNA binding site of the target gene and its carboxy-terminal zinc finger motif. [44, 45] Several studies have reported that MAZ binds to the G4s structure of the transcriptional regulatory region of oncogenes and regulates the transcriptional activity of oncogenes. [32, 38, 46, 47] G4s are guanine-rich secondary structures that differ from the classical DNA double helix, also known as quadruhelix DNA. G4s often appear in the DNA region of pro-oncogenic genes (such as KRAS, E2F, and MYC), and play important roles in the regulation of gene expression, telomere synthesis, and gene recombination. [48] Interestingly, the GSEA enrichment analysis indicated that FHL3 potentially regulates the E2F, KRAS, and MYC signaling pathways (Fig. 5B). [39, 49, 50] CUT&Tag also revealed that FHL3 knockout affected the binding of MAZ to the G4s structure of the KRAS promoter region. Therefore, we speculate that FHL3 may promote the transcriptional function of the MAZ by stabilizing this temporary spatial structure.

KRAS belongs to the RAS superfamily, and the protein it encodes is a small GTPase. For decades, researchers have identified KRAS as an important therapeutic target for cancers. [51, 52] Although RAS mutations are infrequent in HCC, the overexpression of wild-type pan-RAS proteins occurs in a variety of human cancers, such as chronic lymphocytic leukemia and breast cancer. [53, 54] In addition, the upregulation of wild-type KRAS in HCC for various reasons also promotes tumor progression and leads to a poor prognosis. [35] In our study, we found that FHL3 recruits MAZ to the G4s structure of the KRAS regulatory sequence, activating KRAS expression. In addition, rescue assays confirmed that FHL3 promoted the progression of HCC mainly by regulating the expression of KRAS. More importantly, we demonstrated the specific molecular mechanism by which FHL3 is regulated by YAP. FHL3 knockdown inhibited the YAP-induced proliferation, invasion, and metastasis of HCC cells (Fig. 8G). These results indicate that FHL3 may be a potential diagnostic and therapeutic target for patients with Hippo-YAP signaling pathway abnormalities.

## Supplementary information

Supplementary material

Original western blots

## Data Availability

The data included in this study are available from the corresponding authors.
