# Supplementary material for: The Hippo-YAP signaling pathway promotes hepatocellular carcinoma progression by inducing FHL3 expression
Source: Cell Death Dis. 2025 Nov 3;16(1):789. doi: 10.1038/s41419-025-08117-7 (PMC12583741; doi:10.1038/s41419-025-08117-7)
Supplement: Supplementary file 1 — Supplementary material [file 41419_2025_8117_MOESM1_ESM.docx]

**The Hippo-YAP signaling pathway promotes hepatocellular carcinoma progression by inducing FHL3 expression**

Dean Rao^1^, Tiantian Wang^1^, Chengpeng Yu^1^, Hanhua Dong^1, 2^, Wei Yan^1^, Chenan Fu^1^, Yiming Luo^1^, Junli Lu^1^, Zhoubing Sun^1^, Huifang Liang^1, 2^, Wenjie Huang^1, 2, *^, Limin Xia^3, 4, *^

**Supplementary material**

**Materials and methods**

**Patients and tissue specimens**

Total RNA and protein were obtained from 50 pairs of liquid nitrogen frozen tissue samples taken from patients undergoing HCC resection at the Liver Surgery Center of Tongji Hospital Affiliated to Huazhong University of Science and Technology from 2019 to 2020. 122 pairs of tissue chip samples were taken from patients who underwent HCC resection at the Liver Surgery Center of Tongji Hospital Affiliated to Huazhong University of Science and Technology from 2012 to 2016. All patient tissue samples used in this study were approved by the Ethics Committee of Tongji Hospital, Huazhong University of Science and Technology.

**Cell line and cell culture**

The cell lines Hep3B (GDC0070), Huh7 (GDC0134) and HEK-293T used in this experiment were purchased from the China Center for Type Culture Collection (CCTCC, Wuhan, China).  HLF, MHCC97H was derived from the Institute of Liver Cancer of Zhongshan Hospital Affiliated to Fudan University in Shanghai.  All cell lines used in the experiment were cultured using Dulbecco’s modified Eagle medium (DMEM) (Hyclone, South Logan, UT, USA) mixed with 10% fetal bovine serum (FBS) (Newzerum, Christchurch, New Zealand). The cells were cultured in a 37℃ constant temperature incubator containing 5% CO2, and the medium was changed or the cells were passed every 24 hours. The cell experiment was performed in accordance with the aseptic principle.

**Transfection of plasmids**

Plasmids PT3-YAP (#86497), PT3-c-MYC (#92046), PT3-AKT (#31789) are purchased from addgene (addgene, MA, USA). The cDNA sequences of human YAP, FHL3, MAZ, and KRAS were selected according to the sequences on the NCBI website (https://www.ncbi.nlm.nih.gov/), purchased from Tsingke Biotech Co., Ltd (Tsingke, Beijing, China) and cloned into pLenti vector (#174848, Addgene, MA, USA). Knockdown sequences were obtained from the pre-designed shRNA service on Sigma's website (https://www.sigmaaldrich.cn), synthesized by Tsingke and inserted into the pLKO.1 vector (#8453, Addgene, MA, USA). The gene knockdown sequences are shown in Supplementary Table S1.

**Cell transfection and infection**

For transient overexpression of genes, plasmids containing target genes were transfected into cells using Lipofectamine 3000 (Invitrogen, Carlsbad, CA, USA), and gene expression was verified by RT-qPCR or Western blot. To construct stable overexpressed and knock-down cell lines, cells were infected with lentiviruses for 48 hours, then screened for two weeks with the appropriate antibiotic based on the resistance expressed by the cells, and gene expression was verified by RT-qPCR or Western blot.

**Total RNA extraction and Real-time quantitative PCR (RT-qPCR)**

Cells or milled tissues were cleaved by TRIzol (Takara, Tokyo, Japan) and total RNA was extracted according to the reagent instructions. Reverse transcription kits (Vazyme, Nanjing, China) are used to remove genomic DNA and obtain cDNA. The gene expression level in cells or tissues was obtained by RT-qPCR kit (Vazyme, Nanjing, China). Three duplicate values were set for each sample. The RT-qPCR primers used in this study are shown in Supplementary Table S2.

**Western blot**

Total proteins were extracted from tissues and cells using RIPA cracking buffers (Boster Biological Technology, Wuhan, China) supplemented with protease inhibitors and phosphatase inhibitors (Roche, Basel, Switzerland). BCA protein quantification kit (Thermo Fisher Scientific, Waltham, MA, USA) was used for protein quantification. The protein was isolated by electrophoresis using 10% acrylamide SDS-PAGE gel (Yeasen, Shanghai, China) and transferred to PVDF membrane (Merck Millipore, MA, USA). The PVDF membrane was sealed with 5% skim milk for 1 hour and then placed in an appropriate concentration of antibody solution overnight under 4℃ conditions. After washing with TBST for three times, the membrane was incubated with HRP labeled goat anti-mouse or anti-rabbit secondary antibody (ab6702, ab205719, 1:2000, Abcam, MA, USA) at room temperature for 1 hour. Visualization using chemiluminescence (ECL) reagents (Merck Millipore, MA, USA), quantification and analysis using Image Lab™4.0 software (Bio-Rad Laboratories).

The primary antibodies used are listed below.

| Antibodies | Source |
| --- | --- |
| anti-FHL3 | Proteintech, #11028-2-AP |
| anti-GAPDH | Proteintech, #60004-1-Ig |
| anti-KRAS | Proteintech, #12063-1-AP |
| anti-STAT3 | Proteintech, #10253-2-AP |
| Anti-p-STAT3 | Proteintech, #28945-1-AP |
| anti-Flag | Sigma, #F1804 |
| anti-HA | Sigma, #F6908 |
| anti-MAZ | Abcam, #ab85725 |
| anti-c-MYC | Selleck #A5011 |
| anti-YAP | Cell Signaling Technology, #14074 |
| anti-ERK1/2 | Cell Signaling Technology, #9102 |
| anti-p-ERK1/2 | Cell Signaling Technology, #4370 |
| anti-AKT | Cell Signaling Technology, #4685 |
| anti-p-AKT | Cell Signaling Technology, #4060 |

**Immunohistochemistry (IHC)**

Fresh cancer and para-cancer tissues removed from surgical patients or experimental animals were washed with phosphate buffered saline (PBS) and soaked in 4% paraformaldehyde for embedding and preparation of sections. Immunohistochemical experiments were carried out according to the recommended steps of the histochemical kit (ZSGB-BIO, Beijing, China), and then appropriate DAB reaction solution was added to the sections and observed under a low-power microscope to determine the incubation time. The article number and concentration of the primary antibody are as follows: anti-FHL3, #11028-2-AP, 1:100, anti-KRAS, 12063-1-AP, 1:500, Proteintech, Wuhan, China, and anti-YAP, #14074, 1:200, CST, MA, USA, anti-c-MYC, #A5011, 1:100, Selleck, TX, USA, and anti-p-AKT, #4060, 1:100, CST, MA, USA). Finally, dehydrated and sealed with neutral gum. The image was collected by slide scanner and processed and analyzed by Image Viewer G software. We invited three experimenters to score the results or calculate the percentage of positive cells without prior knowledge of the image information.

**Proliferation assay in vitro**

An appropriate number of cells were seeded in 24-well plates and cultured overnight until the cells returned to normal state. Meilun EdU Cell Proliferation Kit with Alexa Fluor 555 (Meilunbio, Dalian, China) was used to detect cell proliferation according to the instructions, and then fluorescence microscopy was used to detect cell proliferation activity and obtain images. For the CCK-8 assay, 1000 cells were planted in 96-well plates, and after the cells were affixed to culture dishes and returned to normal state, CCK-8 enhanced solution (keruibio, Wuhan, China) was co-incubated with the cells for 90 minutes. The absorbance at 450nm (A450) was measured using an ELx 800 Universal Microplate Reader (BioTek, Winooski, VT, USA). There were 6 replicates in each group. Each experiment was repeated three times.

**Wound healing and Transwell Assays**

For wound healing tests, a 200µl pipette tip was used to create scratches in six-well plates with HCC cells cultured. Phase contrast microscopy (Nikon Digital ECLIPSE C1 system, Nikon Corporation) was used to record the migration distance of the cells at 0,24 and 36h. Cell migration and invasion experiments were performed using 24-well transwell plates (8μm pore size, Jet Bio-Fil, China). For migration experiments, a medium containing 10% fetal bovine serum was added to the lower chamber and a serum-free medium containing HCC cells was added to the upper chamber. After 24 hours of cell culture, the remaining cells in the upper chamber were gently wiped with a cotton swab, while the cells on the lower surface were retained. The cells on the lower surface of the chamber were fixed with 4% paraformaldehyde and stained with crystal violet, then the images were taken with a microscope and the number of cells passing through the chamber was counted. For invasion assays, before adding a serum-free medium containing HCC cells to the chamber, the upper part of the chamber was precoated with a mixture of 50μl matrix gel and DMEM. Each experiment was repeated three times.

**Co-Immunoprecipitation (Co-IP)**

After the supernatant in the cell culture dish was discarded and washed with pre-cooled PBS, the cells were lysed with IP lysis containing protease inhibitors and phosphatase inhibitors and collected into 1.5ml centrifuge tubes. After ultrasonic treatment and centrifugation, the supernatant was taken, of which 30 microliter supernatant was used as Input. The remaining supernatants were pre-adsorbed by agarose beads and divided into two parts, adding IgG antibody and target antibody respectively, and incubated in a shaker at 4 degrees Celsius overnight. On the second day, the supernatant containing antibodies was co-incubated in agarose beads for 2h, and then the agarose beads were washed with IP lysis once and IP wash for 5-6 times. Finally, 30 microliter of 2 * loading buffer was added into the centrifuge tube and boiled for silver staining experiment (Beyotime, Shanghai, China), WB experiment or mass spectrometry detection (PTM Bio, Hangzhou, China).

**NanoBiT Protein-Protein Interaction System Assay**

The NanoBiT® PPI MCS Starter System is purchased from Promega (#N2014). First, the pBiT1.1-C [TK/LgBiT] FHL3 and pBiT2.1-C [TK/SmBiT] MAZ plasmids were constructed. The EcoRI and XhoI cleavage sites were used for homologous recombination. The primers with homologous arms used for PCR are as follows: pcr-FHL3-F: agtgggagctcaggggaattcATGAGCGAGTCATTTGACTGTGC; pcr-FHL3-R: ctcccgccaccaccgctcgagGGGCCCTGCCTGGCTACA; pcr-MAZ-F: agtgggagctcagggg

aattcATGTTCCCGGTGTTTCCTTGC; pcr-MAZ-R: ctcccgccaccaccgctcgagCCAGG

GTTGGGAGGGAAGT. The NanoBiT expression vector with HSV-TK promoter was transfected instantaneously in 96-well plates. The cells were incubated in a 5% CO2 incubator at 37℃ for 24 hours. Restore the Nano-Glo LCS Dilution Buffer to room temperature. 1 volume of Nano-Glo Live Cell Substrate was mixed with 19 volume of Nano-Glo LCS Dilution Buffer to produce 5x reserve solution for addition to cell culture medium. Prior to the start of the experiment, the existing cell medium was extracted from the 96-well plate and replaced with 100μl Opti-MEM cell medium, and the 96-well plate was placed in 37℃ incubator for 10 minutes. Add 25μl Nano-Glo Live Cell Reagent to each well and gently mix the plate manually. Finally, the chemiluminescence signal was detected by a multifunctional enzyme marker (Varioskan, Tongji Hospital scientific research public platform).

**Immunofluorescence and laser confocal experiments**

The pancreatic digested cells were transferred into confocal dishes and cultured until they returned to normal state. The cells were fixed with 4% paraformaldehyde, then the membranes were broken with 0.5% Triton X. Samples were sealed with PBS containing 5% BSA at room temperature for 1 hour and incubated with the target antibody at 4℃ overnight. The next day, Sample were cleaned three times with PBS and incubated at room temperature for 1 hour with fluorescent secondary antibody. The nucleus was stained with DAPI and finally sealed with a quench agent. The sample was photographed using a confocal laser microscope in Experimental Medicine Research Center, Tongji Hospital, Wuhan (Olympus FV1000, Tokyo, Japan).

**Molecular docking experiment**

The molecular docking experiment was commissioned by shuli.shop Technology Co., LTD. The Protein structures of MAZ and FHL3 were obtained by AlphaFold Protein Structure Database, and further optimized by protein-protein Docking scheme of Rosetta 3.12. HDock server was used for restricted global docking and Rosetta Interface Analyzer protoco module was used to evaluate the binding area of the two protein complexes and the binding energy of the interaction interface. By modeling the structure prediction of the G4s sequence and 10 ns optimization of the modeled G4s through MD scheme, the conformation with the lowest Gibbs free energy is finally selected as the G4s structure of the sequence, and HDock server was further used for the global docking of MAZH-FHL3 and G4s.

**CUT&Tag experiment**

Protein-DNA interactions were detected using Hyperactive Universal CUT&Tag Assay Kit for Illumina (Vazyme, Nanjing, China). Each group prepared 100,000 cells and carried out experiments according to the kit instructions. The specific experimental products were verified by DNA electrophoresis and tested by Novogene Sequencing company (Novogene, Beijing, China).

**Chromatin immunocoprecipitation (ChIP)**

Cells were cultured in 15cm cell culture dishes, and subsequent operations were carried out when the cell density reached 80%. The experimental products were obtained according to the protocol operation of the 56383 SimpleChIP(R) Plus Sonication ChIP Kit 4C and RT Reagents (CST, MA, USA), and quantitative analysis of the experimental products was performed by RT-qPCR. The antibodies used in the experiment include anti-FHL3, #11028-2-AP, Proteintech, Wuhan, China, anti-MAZ, #ab85725, Abcam, MA, USA, anti-1H6, #MABE1126, Sigma, MO, USA, and anti-YAP, #14074, CST, MA, USA. Primers for KRAS G4s (F: 5′-GTACGCCCGTCTGAAGAAGA-3′; R: 5′-GAGCACACCGATGAGTTCGG-3′) and Distal primer 1 (F: 5′-GAGACTGTCTTAGGTGCTGGAAATT-3′; R: 5′-CTCCAGAG

CTCTCATCACCTTCA-3′). Primers for FHL3 YAP/TEAD binding site (F: 5′-AGGTCCCCTCCCCTCTCCTC-3′; R: 5′-TGACACTGAGAAGTCCTGCCTTGC-3′) and Distal primer 2 (F: 5′-TCCTCTTGGTACCCACTCCACC-3′; R: 5′-CTCCCAAAGTGCTGGGACCAC-3′).

**Luciferase reporter assay**

Firstly, human genomic DNA was extracted from the blood using a DNA extraction kit (Vazyme, Nanjing, China). PCR primers were designed with reference to FHL3 gene regulatory sequence and target fragments were obtained by PCR. pGL4.17 plasmid was used as the vector to insert the fragment. The cells were inoculated in 24-well plates and transfected when cells were attached to the wall and returned to normal state. 12 hours after transfection, the fluid was changed, and 48 hours after the fluid was changed, the experimental operation was carried out using the double luciferase reporter kit (Promega, WI, USA), and then the fluorescence value of firefly and sea kidney were respectively detected by the machine and the ratio between the two was calculated as the final experimental results.

**Supplementary Figure S1**


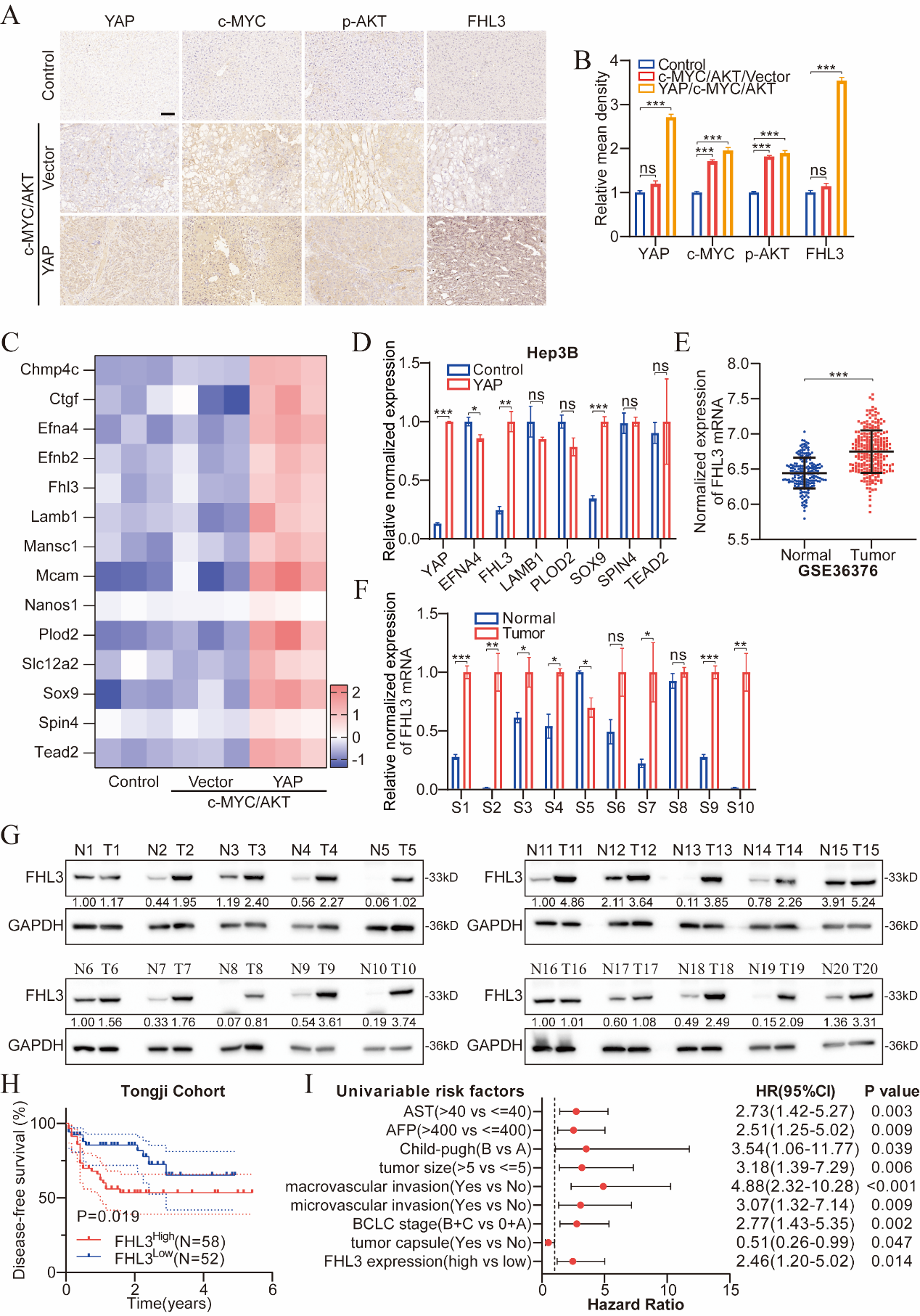


**Figure S1: FHL3 is highly expressed in hepatocellular carcinoma and is associated with poor prognosis.**

(A) Immunohistochemical staining showed the expression of YAP, c-MYC, p-AKT and FHL3 in spontaneous HCC mouse tissues. Scale bar:50μm.

(B) Statistical diagram of YAP, c-MYC, p-AKT and FHL3 immunohistochemical staining.

(C) 14 candidate genes obtained through Venn diagram analysis.

(D) The downstream candidate genes of YAP were screened by RT-qPCR.

(E) The expression level of FHL3 in cancer tissues and adjacent tissues in GSE36376.

(F) RT-qPCR showed the expression of FHL3 mRNA in 10 of 50 pairs of cancer and adjacent tissues.

(G) WB showed the expression of FHL3 protein in 20 of 50 pairs of cancer and adjacent tissues.

(H) The Kaplan-Meier plots of the DFS rates of groups with FHL3 differential expression in Tongji cohort.

(I) The Forest plot of the univariate Cox proportional hazards model for overall survival of FHL3. Data are represented as mean ± SEM. ns: not significant, *: P < 0.05, **: P < 0.01, ***: P < 0.001.

**Supplementary Figure S2**


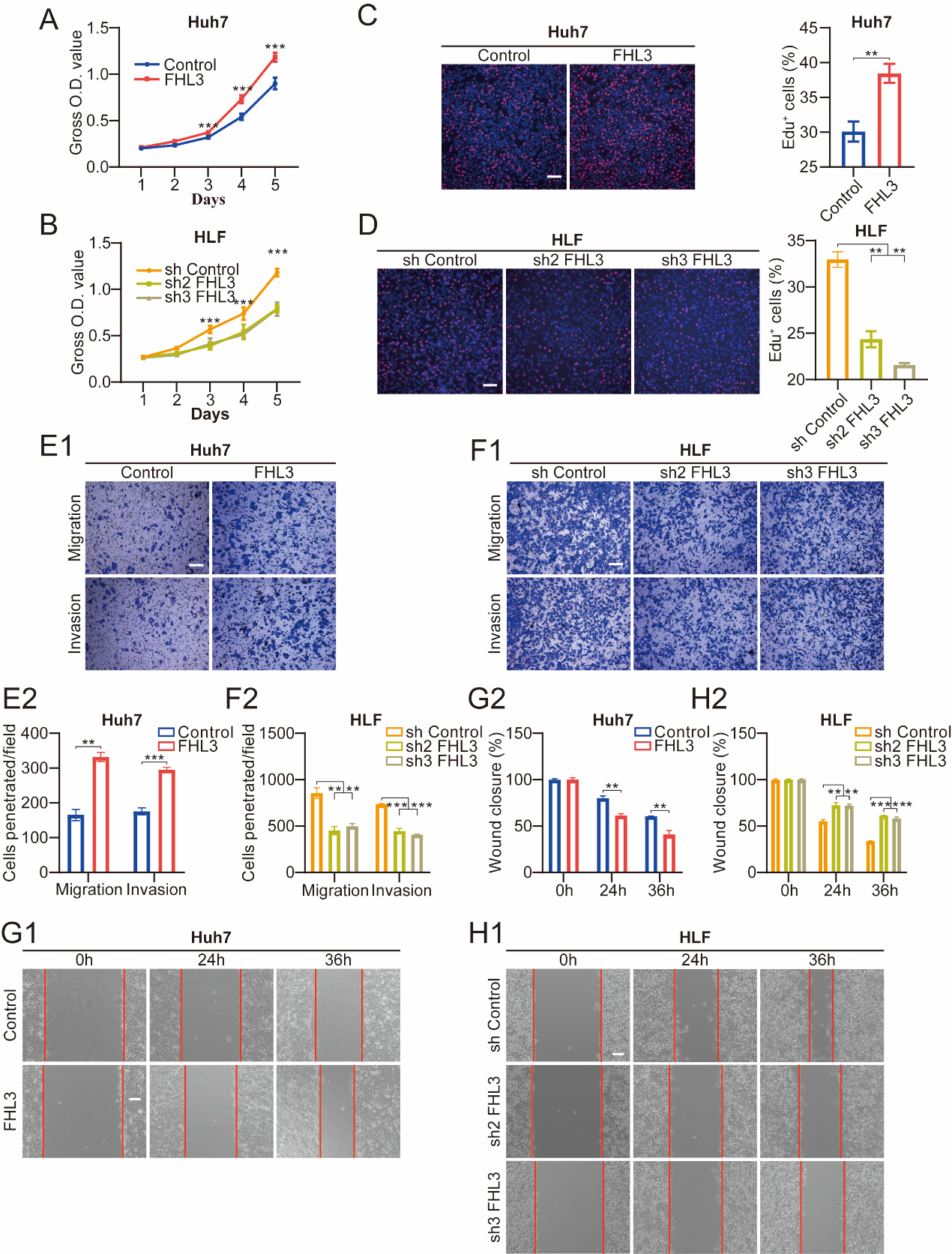


**Figure S2: FHL3 promotes the proliferation, invasion and migration of HCC cells in vitro.**

(A) CCK8 assay in Huh7 cell line with stable overexpression of FHL3. Each experiment was repeated three times.

(B) CCK8 assay in HLF cell line with stable knockdown of FHL3.

(C) Representative images of EdU incorporation assay in Huh7 cell line with stable overexpression of FHL3. Scale bar:100μm. Each experiment was repeated three times.

(D) Representative images of EdU incorporation assay in HLF cell line with stable knockdown of FHL3. Scale bar:100μm.

(E) Representative images of Transwell assay in Huh7 cell line with stable overexpression of FHL3 (E1) and corresponding statistical graph (E2). Scale bar: 100μm. Each experiment was repeated three times.

(F) Representative images of Transwell assay in HLF cell line with stable knockdown of FHL3 (F1) and corresponding statistical graph (F2). Scale bar: 100μm.

(G) Representative images of Scratch assay in Huh7 cell line with stable overexpression of FHL3 (G1) and corresponding statistical graph (G2). Scale bar: 100μm. Each experiment was repeated three times.

(H) Representative images of Scratch assay in HLF cell line with stable knockdown of FHL3 (H1) and corresponding statistical graph (H2). Scale bar: 100μm. Data are represented as mean ± SEM. ns: not significant, *: P < 0.05, **: P < 0.01, ***: P < 0.001.

**Supplementary Figure S3**


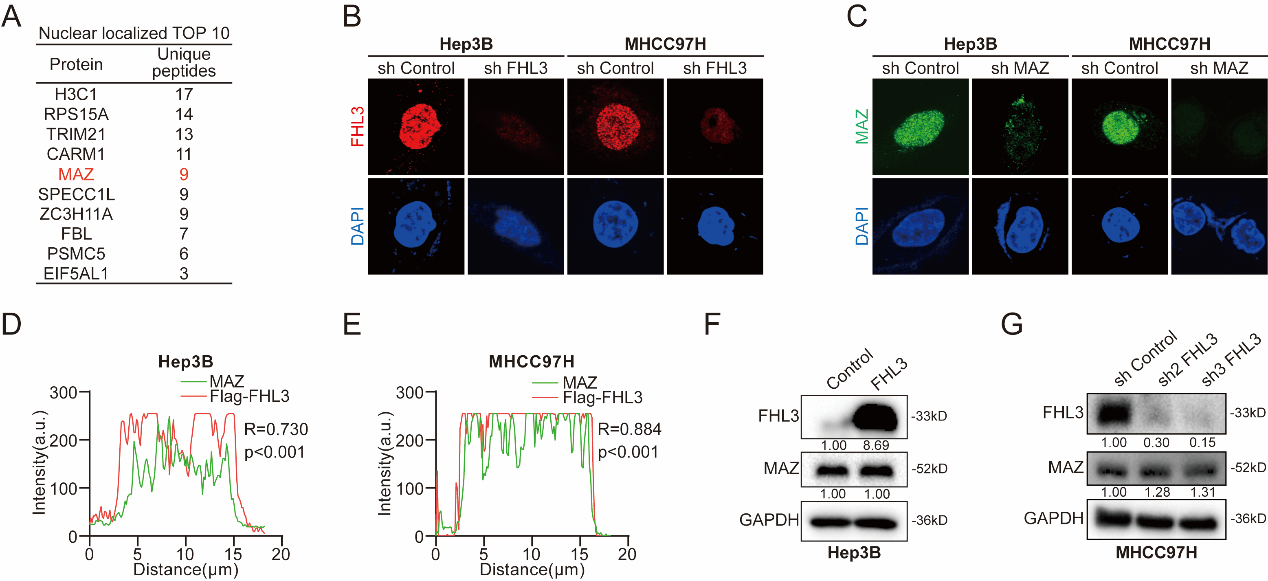


**Figure S3: Quantitative** **correlation analysis of fluorescence value of confocal immunofluorescence.**

(A) The top 10 nuclear-localized proteins from the MS candidate proteins

(B) The suitability of FHL3 antibody was tested by immunofluorescence assay.

(C) The suitability of MAZ antibody was tested by immunofluorescence assay.

(D) Quantitative correlation analysis of fluorescence values of MAZ and Flag-FHL3 in Hep3B.

(E) Quantitative correlation analysis of fluorescence values of MAZ and Flag-FHL3 in MHCC97H.

(F) WB experiment showed that overexpression of FHL3 did not affect the protein level of MAZ in Hep3B.

(G) WB experiment showed that FHL3 knockdown in MHCC97H did not affect the protein level of MAZ.

**Supplementary Figure S4**


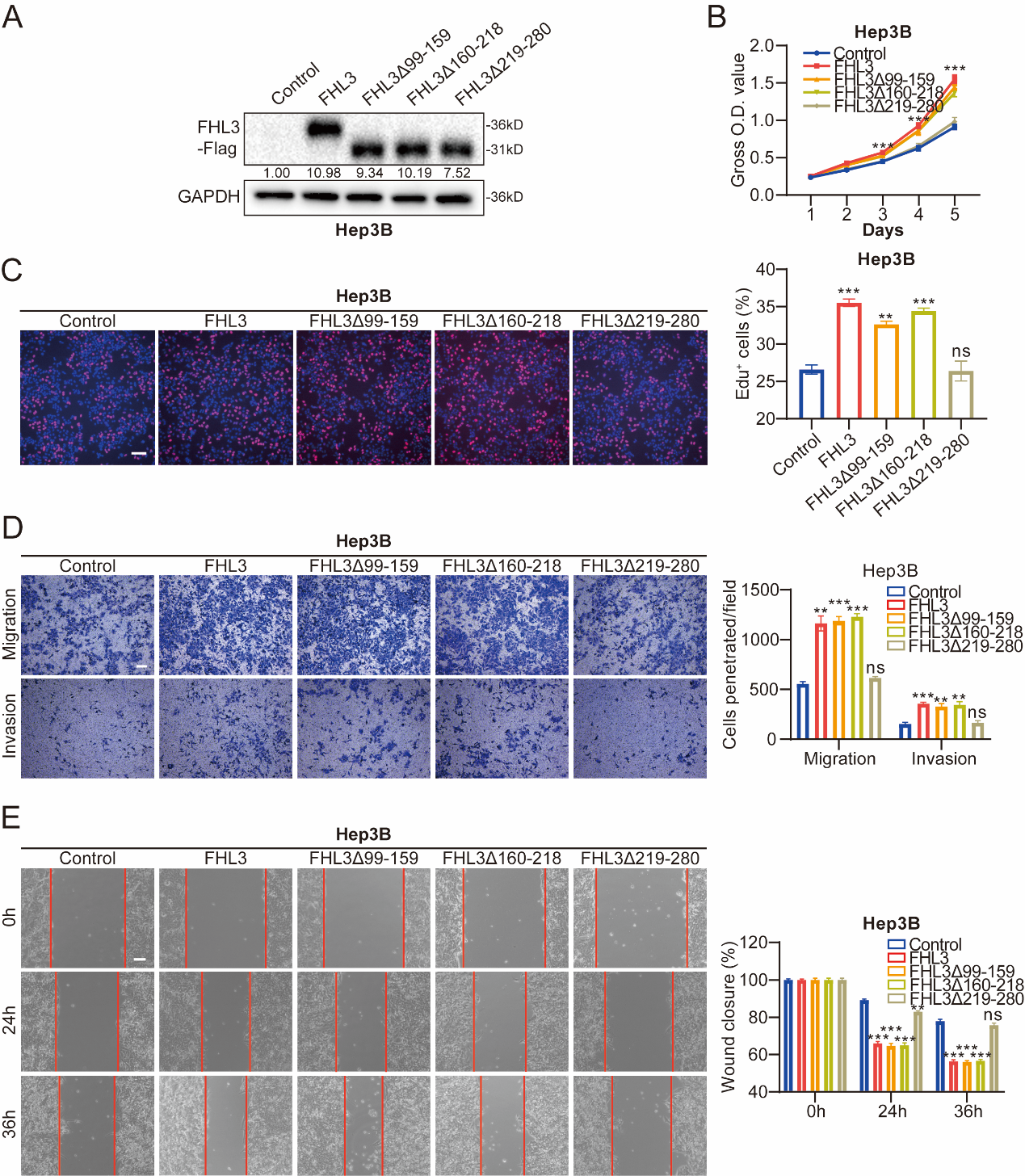


**Figure S4: The LIM4 domain of FHL3 is necessary for the carcinogenic effect of FHL3.**

(A) WB indicates the absence of the FHL3-Flag domain.

(B) CCK8 assay showed the influence of FHL3 domains on the proliferation of Hep3B cell line. Each experiment was repeated three times.

(C) Representative images of EdU incorporation experiments show the effects of FHL3 domains on the proliferation of Hep3B cell lines (left) and corresponding statistical maps (right). Each experiment was repeated three times. Scale bar: 100μm.

**(D)** Representative images of transwell experiments show the effects of FHL3 domains on the invasion and migration of Hep3B cell lines (left) and corresponding statistical maps (right). Each experiment was repeated three times. Scale bar: 100μm.

**(E)** Representative images of scratch experiments show the effects of FHL3 domains on the migration of Hep3B cell lines (left) and corresponding statistical maps (right). Each experiment was repeated three times. Scale bar: 100μm. Data are represented as mean ± SEM. ns: not significant, *: P < 0.05, **: P < 0.01, ***: P < 0.001.

**Supplementary Figure S5**


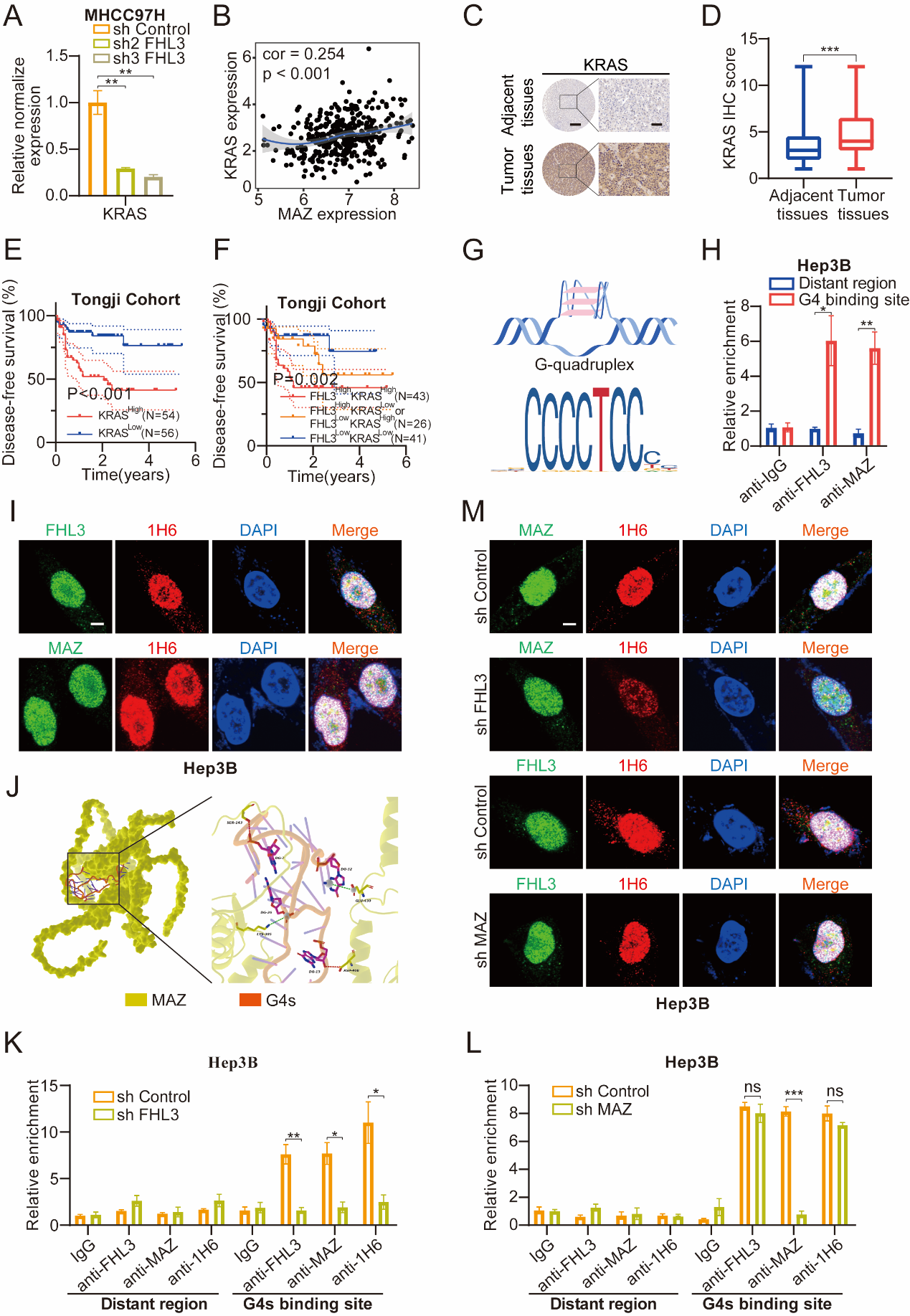


**Figure S5: FHL3 regulates the transcriptional activity of MAZ by influencing the binding of MAZ to G4s.**

(A) qPCR experiments showed the effects of FHL3 or MAZ knockdown on KRAS transcription levels.

(B) The correlation between MAZ and KRAS was calculated based on LIHC expression matrix in TGCA database.

(C) Representative images of KRAS expression levels detected by immunohistochemical staining. Scale bar: overview images, 50μm; magnified images, 200μm.

(D) The expression of KRAS in cancer tissues and adjacent tissues measured by immunohistochemical staining.

(E) The Kaplan-Meier plots of the DFS rates of groups with KRAS differential expression in Tongji cohort.

(F) The Kaplan-Meier plots of the DFS rates of groups with FHL3 and KRAS differential expression in Tongji cohort.

(G) G4s DNA structure schematic (top) and potential MAZ binding sequence (bottom). (H) ChIP experiments showed that FHL3 and MAZ bind to the G4s DNA region of the KRAS promoter in wild-type Hep3B cell line.

(I) Immunofluorescence showed that FHL3 or MAZ proteins were co-located with the G4 DNA region of KRAS promoter in wild-type Hep3B cell line. Scale bar: 5μm.

(J) Molecular docking experiments showed the binding mode of MAZ and G4s in the absence of FHL3.

(K) ChIP experiments showed that FHL3 knockdown in Hep3B cell lines resulted in structural changes in the G4s DNA region of the KRAS promoter (the G4s DNA structure is not recognized by anti-1H6 antibodies), thus preventing MAZ from binding to this site. Scale bar: 5μm.

(L) ChIP experiments showed that MAZ knockdown in Hep3B cell lines had little effect on the structure of the G4s DNA region of the KRAS promoter or its binding to FHL3.

(M). Immunofluorescence revealed loss of colocalization of the MAZ protein and the G4s DNA region of KRAS promoter in the Hep3B cell line with FHL3 knockdown (top). However, the co-localization of the FHL3 protein and the G4s DNA region of KRAS promoter was not affected in the case of MAZ knockdown (bottom). Data are represented as mean ± SEM. ns: not significant, *: P < 0.05, **: P < 0.01, ***: P < 0.001.

**Supplementary Figure S6**


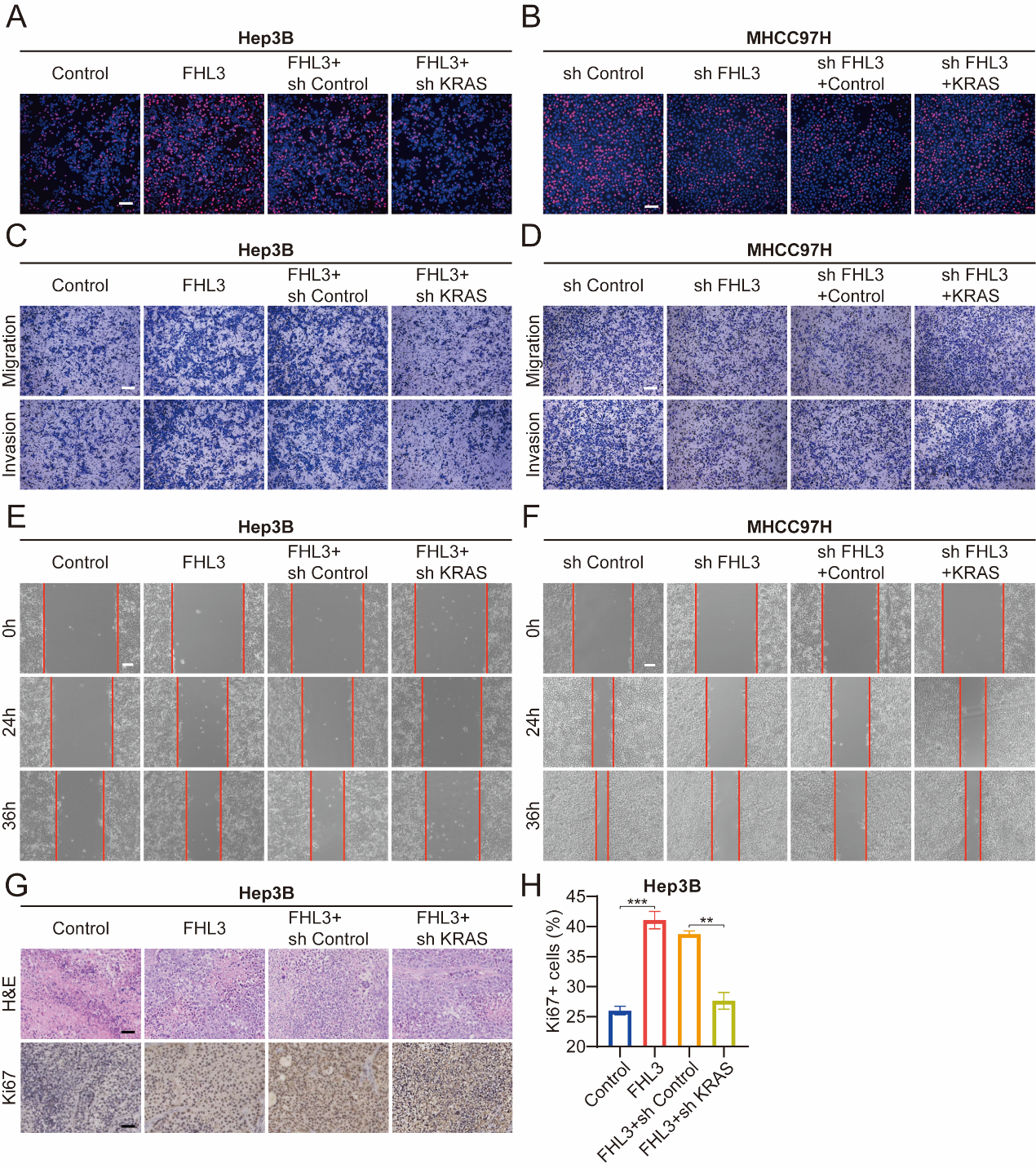


**Figure S6: FHL3 promotes HCC progression through KRAS signaling pathway.**

(A-B) Representative images of EdU experiments based on FHL3 overexpression and KRAS knockdown in Hep3B cell line (A) or KRAS overexpression on the basis of FHL3 knockdown in MHCC97H cell line (B). Each experiment was repeated three times. Scale bar: 100μm.

(C-D) Representative images of transwell assays based on FHL3 overexpression and KRAS knockdown in Hep3B cell line (C) or KRAS overexpression on the basis of FHL3 knockdown in MHCC97H cell line (D). Each experiment was repeated three times. Scale bar: 100μm.

(E-F) Representative images of scratch assays based on FHL3 overexpression and KRAS knockdown in Hep3B cell line (E) or KRAS overexpression on the basis of FHL3 knockdown in MHCC97H cell line (F). Each experiment was repeated three times. Scale bar: 100μm.

(G) Representative images of H&E staining and immunohistochemical staining of subcutaneous tumor tissue implanted with Hep3B cell line. Each experiment was repeated three times. Scale bar: 50μm.

(H) Statistics of Ki67 positive rate of cells. n = 6. Data are represented as mean ± SEM. ns: not significant, *: P < 0.05, **: P < 0.01, ***: P < 0.001.

**Supplementary Figure S7**


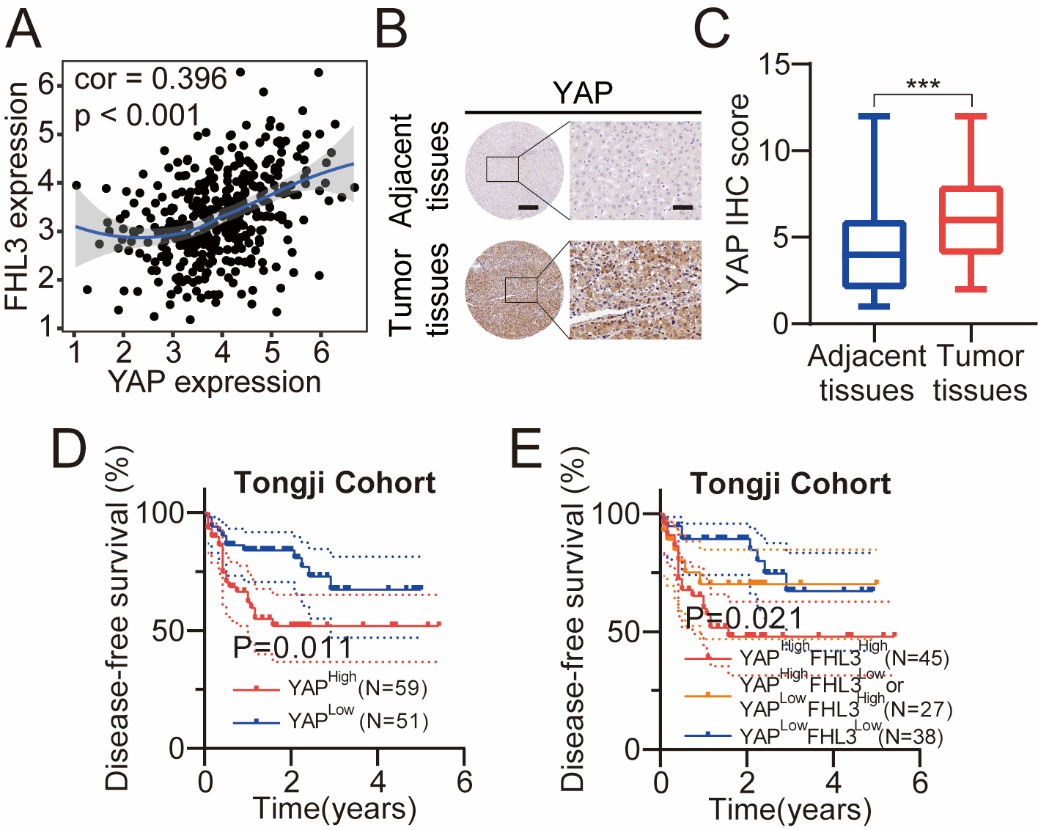


**Figure S7: Correlation between YAP and FHL3 expression.**

(A) The correlation between YAP and FHL3 was calculated based on LIHC expression matrix in TGCA database.

(B) The expression of YAP in cancer tissues and adjacent tissues measured by immunohistochemical staining.

(C) Statistics of YAP expression in cancer tissues and adjacent tissues.

(D) The Kaplan-Meier plots of the DFS rates of groups with YAP differential expression in Tongji cohort.

(E) The Kaplan-Meier plots of the DFS rates of groups with YAP and FHL3 differential expression in Tongji cohort.

**Supplementary Figure S8**


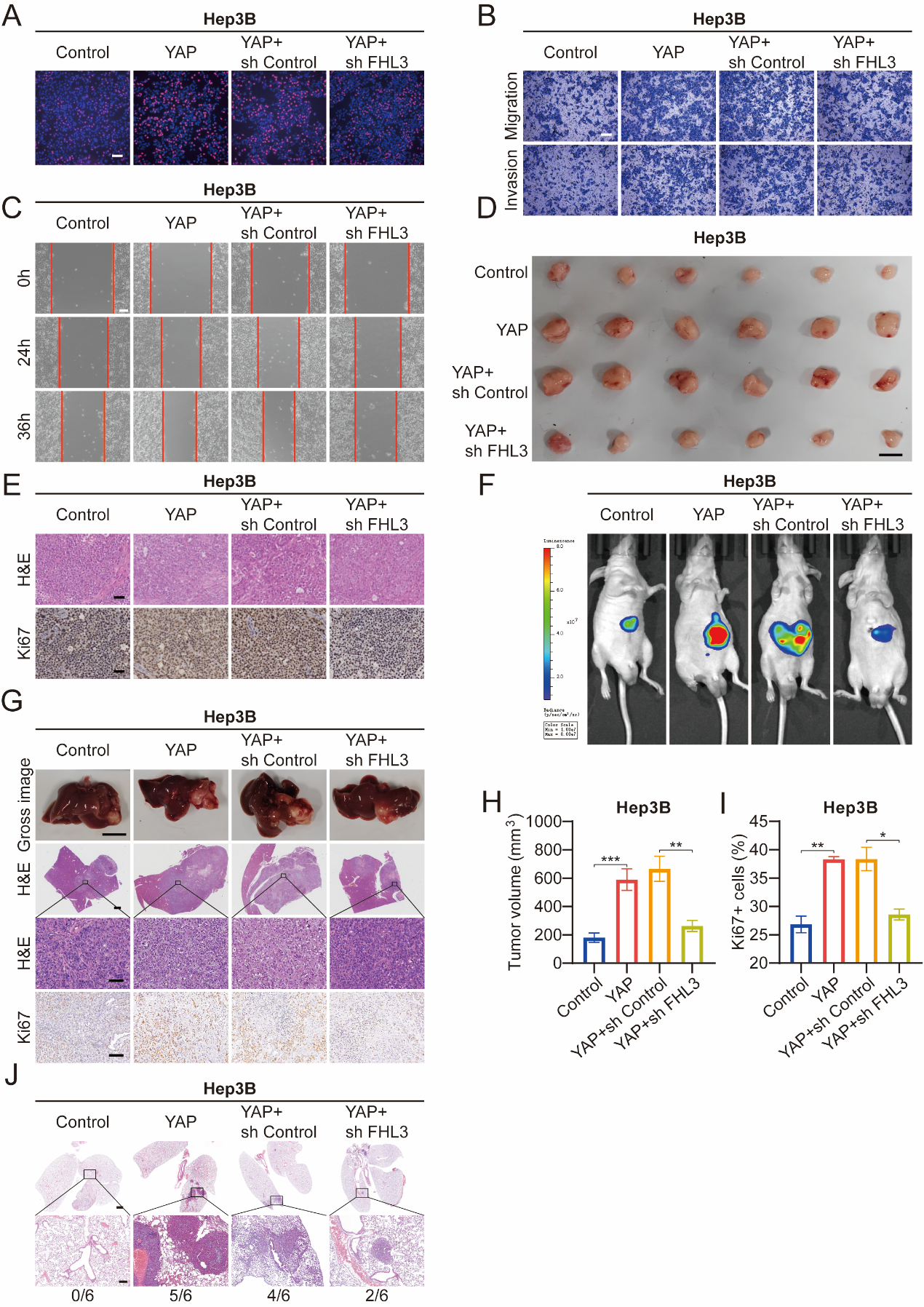


**Figure S8: FHL3 knockdown significantly inhibited the promotion effect of Hippo-YAP signaling pathway on HCC.**

(A) Representative images of EdU experiments based on YAP overexpression and FHL3 knockdown in Hep3B cell line. Each experiment was repeated three times. Scale bar: 100μm.

(B) Representative images of transwell assays based on YAP overexpression and FHL3 knockdown in Hep3B cell line. Each experiment was repeated three times. Scale bar: 100μm.

(C) Representative images of scratch assays based on YAP overexpression and FHL3 knockdown in Hep3B cell line. Each experiment was repeated three times. Scale bar: 100μm.

(D) Subcutaneous tumor implantation experiment with FHL3 knockdown based on overexpression of YAP in Hep3B cell line. n = 6. Scale bar: 1cm.

(E) Representative images of H&E staining and immunohistochemical staining of subcutaneous tumor tissue implanted with Hep3B cell line. Scale bar: 50μm.

(F) Representative images of mouse imaging in situ implantation model of FHL3 knockdown on the basis of YAP overexpression in Hep3B cell line. n = 6.

(G) Representative images of Liver (Scale bar: 1cm), H&E staining of overview images (Scale bar: 1mm), magnified images (Scale bar: 100μm) and Ki67 immunohistochemical staining (Scale bar: 100μm).

(H) Statistics of tumor volume in situ implantation model of Hep3B cell line.

(I) Statistics of proportion of Ki67 in situ implantation model of Hep3B cell line.

(J) Representative images of lung H&E staining and lung metastasis statistics from in situ implantation models of FHL3 knockdown on the basis of YAP overexpression in Hep3B cell line. Scale bar: overview images, 1 mm; magnified images, 200 μm.

**Supplementary Figure S9**


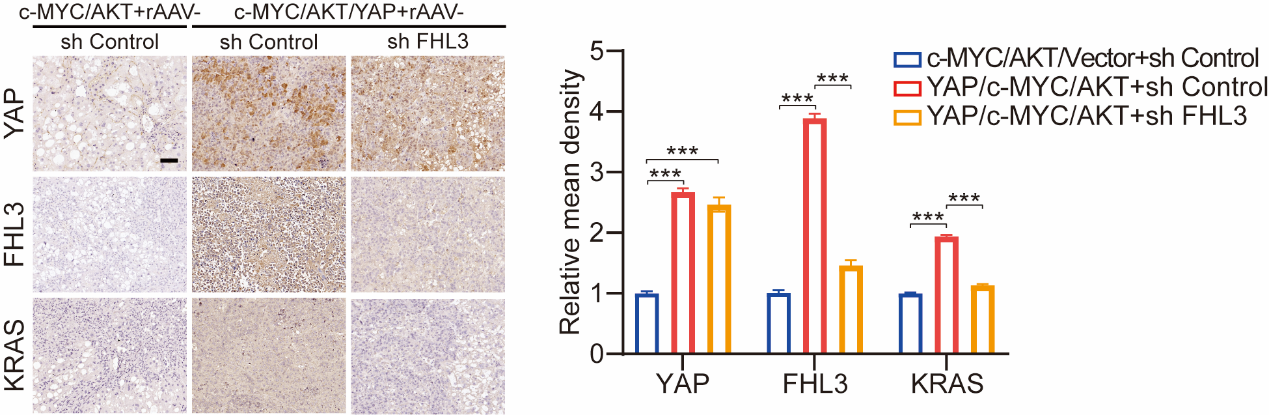


**Figure S9:** Immunohistochemical staining (left) and corresponding statistical graphs (right) showed the expression of YAP, FHL3 and KRAS in spontaneous HCC tissues. Scale bar:50μm. Data are represented as mean ± SEM. ns: not significant, *: P < 0.05, **: P < 0.01, ***: P < 0.001.

Supplementary Table S1: Knockdown shRNA sequences used in this study

| Gene name | Sequence |
| --- | --- |
| shFHL3-1F | CCGGGAGAACTCTTTGCACCTAAGTCTCGAGACTTAGGTGCAAAGAGTTCTCTTTTTG |
| shFHL3-1R | AATTCAAAAAGAGAACTCTTTGCACCTAAGTCTCGAGACTTAGGTGCAAAGAGTTCTC |
| shFHL3-2F | CCGGGCTATGACAATACCTTTGCCACTCGAGTGGCAAAGGTATTGTCATAGCTTTTTG |
| shFHL3-2R | AATTCAAAAAGCTATGACAATACCTTTGCCACTCGAGTGGCAAAGGTATTGTCATAGC |
| shFHL3-3F | CCGGGCAAGTACATCCAGACAGACACTCGAGTGTCTGTCTGGATGTACTTGCTTTTTG |
| shFHL3-3R | AATTCAAAAAGCAAGTACATCCAGACAGACACTCGAGTGTCTGTCTGGATGTACTTGC |
| shMAZ-1F | CCGGGATGCTGAGCTCGGCTTATATCTCGAGATATAAGCCGAGCTCAGCATCTTTTTG |
| shMAZ-1R | AATTCAAAAAGATGCTGAGCTCGGCTTATATCTCGAGATATAAGCCGAGCTCAGCATC |
| shMAZ-2F | CCGGTCTGTGAGCTCTGCAACAAAGCTCGAGCTTTGTTGCAGAGCTCACAGATTTTTG |
| shMAZ-2R | AATTCAAAAATCTGTGAGCTCTGCAACAAAGCTCGAGCTTTGTTGCAGAGCTCACAGA |
| shMAZ-3F | CCGGGCCTTGGAGAAGAAGACAAAGCTCGAGCTTTGTCTTCTTCTCCAAGGCTTTTTG |
| shMAZ-3R | AATTCAAAAAGCCTTGGAGAAGAAGACAAAGCTCGAGCTTTGTCTTCTTCTCCAAGGC |
| shKRAS-1F | CCGGCAGTTGAGACCTTCTAATTGGCTCGAGCCAATTAGAAGGTCTCAACTGTTTTTG |
| shKRAS-1R | AATTCAAAAACAGTTGAGACCTTCTAATTGGCTCGAGCCAATTAGAAGGTCTCAACTG |
| shKRAS-2F | CCGGGAGGGCTTTCTTTGTGTATTTCTCGAGAAATACACAAAGAAAGCCCTCTTTTTG |
| shKRAS-2R | AATTCAAAAAGAGGGCTTTCTTTGTGTATTTCTCGAGAAATACACAAAGAAAGCCCTC |
| shKRAS-3F | CCGGTAGTTGGAGCTGGTGGCGTAGCTCGAGCTACGCCACCAGCTCCAACTATTTTTG |
| shKRAS-3R | AATTCAAAAATAGTTGGAGCTGGTGGCGTAGCTCGAGCTACGCCACCAGCTCCAACTA |

Supplementary Table S2: Primer sequences used in the study

| Primers for RT-PCR | Sequence |
| --- | --- |
| GAPDH-F | 5’-GGAGCGAGATCCCTCCAAAAT-3’ |
| GAPDH-R | 5’-GGCTGTTGTCATACTTCTCATGG-3’ |
| YAP-F | 5’-TGACCCTCGTTTTGCCATGA-3’ |
| YAP-R | 5’-ACCATCCTGCTCCAGTGTTG-3’ |
| FHL3-F | 5’-GAGTCCCTGTATGGACGCAA-3’ |
| FHL3-R | 5’-GCATGACAGTCTCCCCACAA-3’ |
| EFNA4-F | 5’-CATTGTCTGCCCCCACTACG-3’ |
| EFNA4-R | 5’-TCCCAACAGGATGGGCTGAC-3’ |
| LAMB1-F | 5’-ACCAAGATGTCCTGAGTGCC-3’ |
| LAMB1-R | 5’-TTCGGCTTTCCTTCTGGCAT-3’ |
| PLOD2-F | 5’-CTCCACCCCTGGAATCCCT-3’ |
| PLOD2-R | 5’-TTTCTGGCCCCCTCCAATAC-3’ |
| SOX9-F | 5’-AGAACAAGCCGCACGTCAAG-3’ |
| SOX9-R | 5’-GAAGATGGCGTTGGGGGAGAT-3’ |
| SPIN4-F | 5’-AAGCCCACTTTCCTCACTCG-3’ |
| SPIN4-R | 5’-GGGAATCTGCCAGTCGTGAA-3’ |
| TEAD2-F | 5’-CAGTGAGGAAGGCAGTGAGG-3’ |
| TEAD2-R | 5’-TGGGAGGTCAGTAGATGGGG-3’ |
| MAZ-F | 5’- GCCTTCCGCGACGTCTACCAC -3’ |
| MAZ-R | 5’- CGCCCGCAGCCGATCCTTC -3’ |
| KRAS-F | 5’-ACAGAGAGTGGAGGATGCTTT-3’ |
| KRAS-R | 5’-TTTCACACAGCCAGGAGTCTT-3’ |

Supplementary Table S3: Expression and prognosis of 14 candidate genes in HCC

| Gene | Normal<br>(n=50) | | | Primary tumor<br>(n=371) | | | p-value | Survival  p-value |
| --- | --- | --- | --- | --- | --- | --- | --- | --- |
|  | low | median | high | low | median | high |  |  |
| **PLOD2** | 5.861 | 16.836 | 33.745 | 0.586 | 23.702 | 86.902 | 1.49E-10 | p<0.001 |
| **EFNA4** | 1.175 | 2.546 | 5.074 | 0.746 | 10.417 | 31.922 | 1.62E-12 | p<0.001 |
| **TEAD2** | 0.909 | 2.716 | 6.544 | 0.158 | 8.398 | 39.68 | 1.62E-12 | p=0.0018 |
| NANOS1 | 0.335 | 0.77 | 1.272 | 0.253 | 1.346 | 3.508 | 1.62E-12 | p=0.49 |
| MCAM | 0.863 | 5.063 | 12.055 | 1.081 | 16.771 | 54.231 | 1.62E-12 | p=0.2 |
| **SPIN4** | 0.106 | 0.358 | 0.716 | 0.028 | 0.582 | 2.634 | 1.62E-12 | p=0.02 |
| **LAMB1** | 2.64 | 11.543 | 20.66 | 2.28 | 17.755 | 70.378 | 1.11E-16 | p=0.0017 |
| CHMP4C | 1.599 | 3.161 | 5.842 | 0.081 | 6.774 | 20.876 | 1.62E-12 | p=0.16 |
| EFNB2 | 0.427 | 2.239 | 4.14 | 0.232 | 3.352 | 11.769 | 1.62E-12 | p=0.31 |
| **SOX9** | 0.524 | 2.15 | 7.029 | 0 | 5.564 | 37.723 | 1.62E-12 | p=0.0057 |
| SLC12A2 | 0.521 | 2.222 | 5.789 | 0.045 | 1.84 | 11.29 | - | p=0.0057 |
| CTGF | 5.05 | 24.638 | 61.081 | 0.817 | 26.104 | 124.323 | 9.97E-01 | p=0.57 |
| MANSC1 | 2.617 | 4.957 | 8.081 | 0.682 | 7.34 | 19.941 | <1E-12 | p=0.23 |
| **FHL3** | 1.571 | 3.731 | 7.808 | 1.25 | 8.704 | 26.399 | 1.62E-12 | p=0.011 |

Supplementary Table S4: Correlation between differential expression of FHL3 and clinical features

|  | **Total** | **High (n=58)** | **Low (n=52)** | **P-value** |
| --- | --- | --- | --- | --- |
| **Sex** |  |  |  |  |
| Female | 20 | 6 | 14 |  |
| Male | 90 | 52 | 38 | **0.024** |
| **Age** |  |  |  |  |
| <=50 | 54 | 25 | 29 |  |
| >50 | 56 | 33 | 23 | 0.185 |
| **Differentiation** |  |  |  |  |
| High | 30 | 15 | 15 |  |
| Moderate/Low | 80 | 43 | 37 | 0.726 |
| **ALT** |  |  |  |  |
| <=40 | 81 | 45 | 36 |  |
| >40 | 29 | 13 | 16 | 0.321 |
| **AST** |  |  |  |  |
| <=40 | 77 | 43 | 34 |  |
| >40 | 33 | 15 | 18 | 0.317 |
| **AFP** |  |  |  |  |
| <=400 | 57 | 30 | 27 |  |
| >400 | 53 | 28 | 25 | 0.983 |
| **HBV** |  |  |  |  |
| absent | 9 | 3 | 6 |  |
| present | 101 | 55 | 46 | 0.224 |
| **Tumor size (cm)** |  |  |  |  |
| <=5 | 40 | 14 | 26 |  |
| >5 | 70 | 44 | 26 | **0.005** |
| **Cirrhosis** |  |  |  |  |
| absent | 30 | 18 | 12 |  |
| present | 70 | 40 | 40 | 0.350 |
| **Tumor number** |  |  |  |  |
| solitary | 85 | 41 | 44 |  |
| Multiple | 25 | 17 | 8 | 0.563 |
| **Vascular invasion** |  |  |  |  |
| absent | 87 | 40 | 45 |  |
| present | 23 | 18 | 7 | **0.028** |
| **BCLC stage** |  |  |  |  |
| 0+A | 71 | 31 | 40 |  |
| B+C | 39 | 27 | 12 | **0.010** |
| **TNM stage** |  |  |  |  |
| I+II | 81 | 41 | 40 |  |
| III+IV | 29 | 17 | 12 | 0.459 |
| **Tumor capsule** |  |  |  |  |
| absent | 50 | 27 | 23 |  |
| present | 60 | 31 | 29 | 0.807 |
| **Recurrence** |  |  |  |  |
| No | 78 | 40 | 38 |  |
| Yes | 32 | 18 | 14 | 0.636 |

Supplementary Table S5: FHL3 and G4s interaction result

| Interaction | FHL3 | G4s | Distance (noH) |
| --- | --- | --- | --- |
| Hydrogen Bond | Phe263 | DG18 | 2.63 |

Supplementary Table S6: MAZ and G4s interaction result (FHL3/MAZ/G4s)

| Interaction | MAZH | G4s | Distance (noH) |
| --- | --- | --- | --- |
| Hydrogen Bond | Ser347 | DG32 | 3.20 |
| Hydrogen Bond | Lys335 | DG9 | 3.16 |
| Salt Bridge | Lys305 | DG15 | 4.74 |
| Salt Bridge | Lys335 | DG32 | 3.99 |
| Salt Bridge | Lys335 | DG9 | 3.97 |
| Salt Bridge | Lys335 | DT10 | 4.98 |

Supplementary Table S7: MAZH and G4s interaction result (MAZ/G4s)

| Interaction | MAZH | G4s | Distance (noH) |
| --- | --- | --- | --- |
| Hydrogen Bond | Asp408 | DG15 | 3.21 |
| Hydrogen Bond | Ser143 | DG7 | 3.22 |
| Salt Bridge | Lys305 | DG26 | 5.19 |
| Salt Bridge | Glu430 | DG32 | 5.30 |
