# Supplementary figures and images for: The Hippo-YAP signaling pathway promotes hepatocellular carcinoma progression by inducing FHL3 expression
Source: Cell Death Dis. 2025 Nov 3;16(1):789. doi: 10.1038/s41419-025-08117-7 (PMC12583741; doi:10.1038/s41419-025-08117-7)

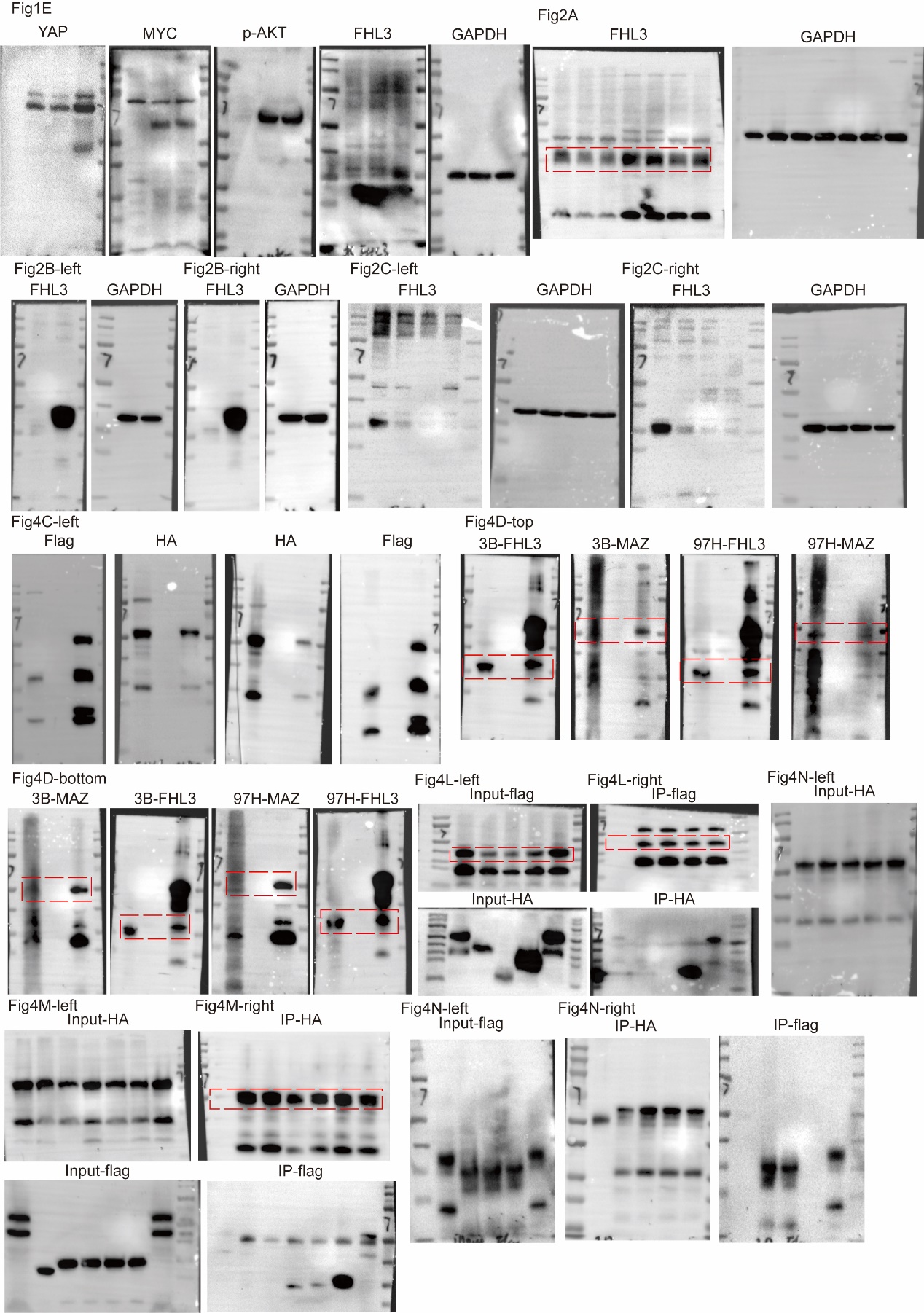

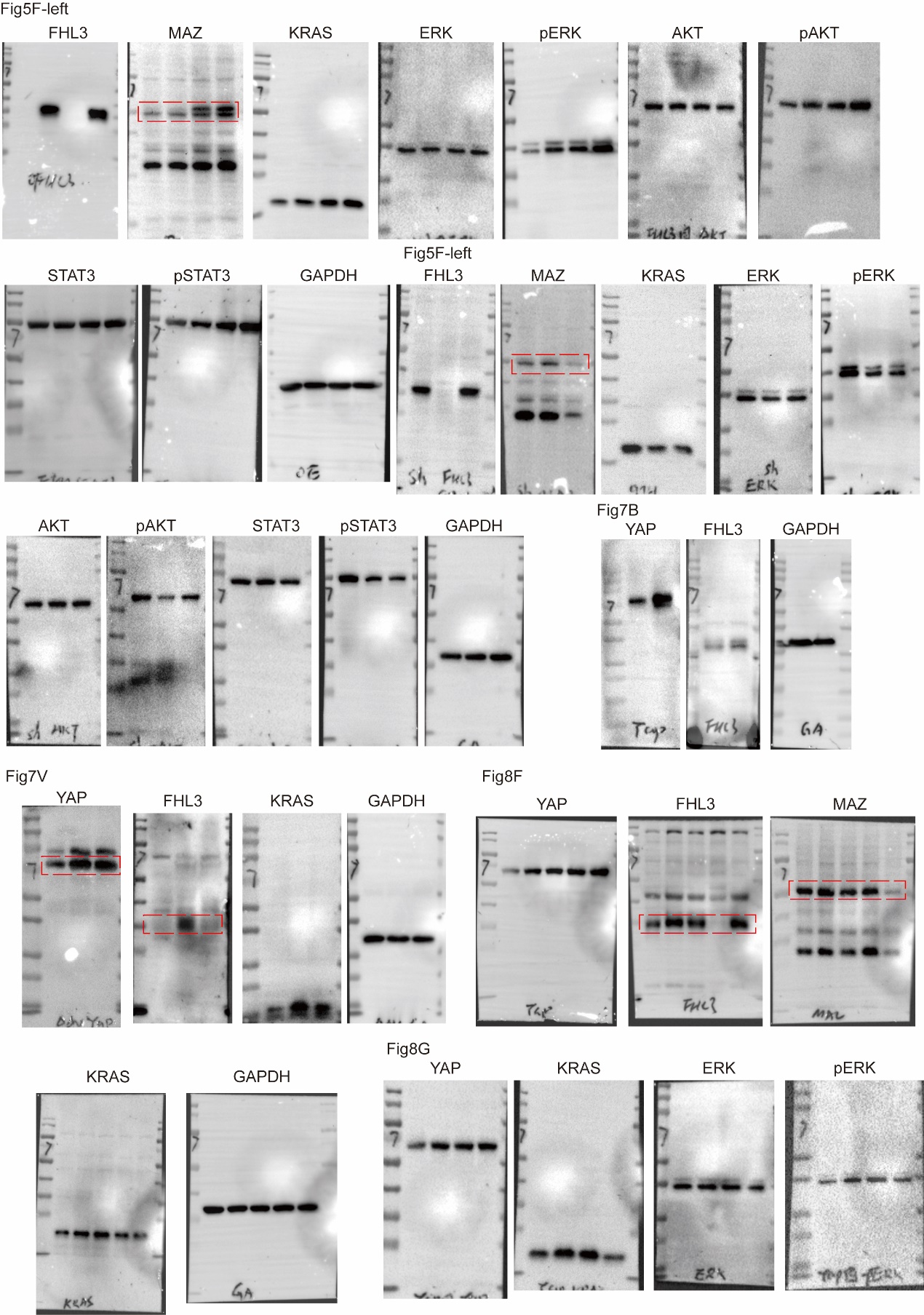

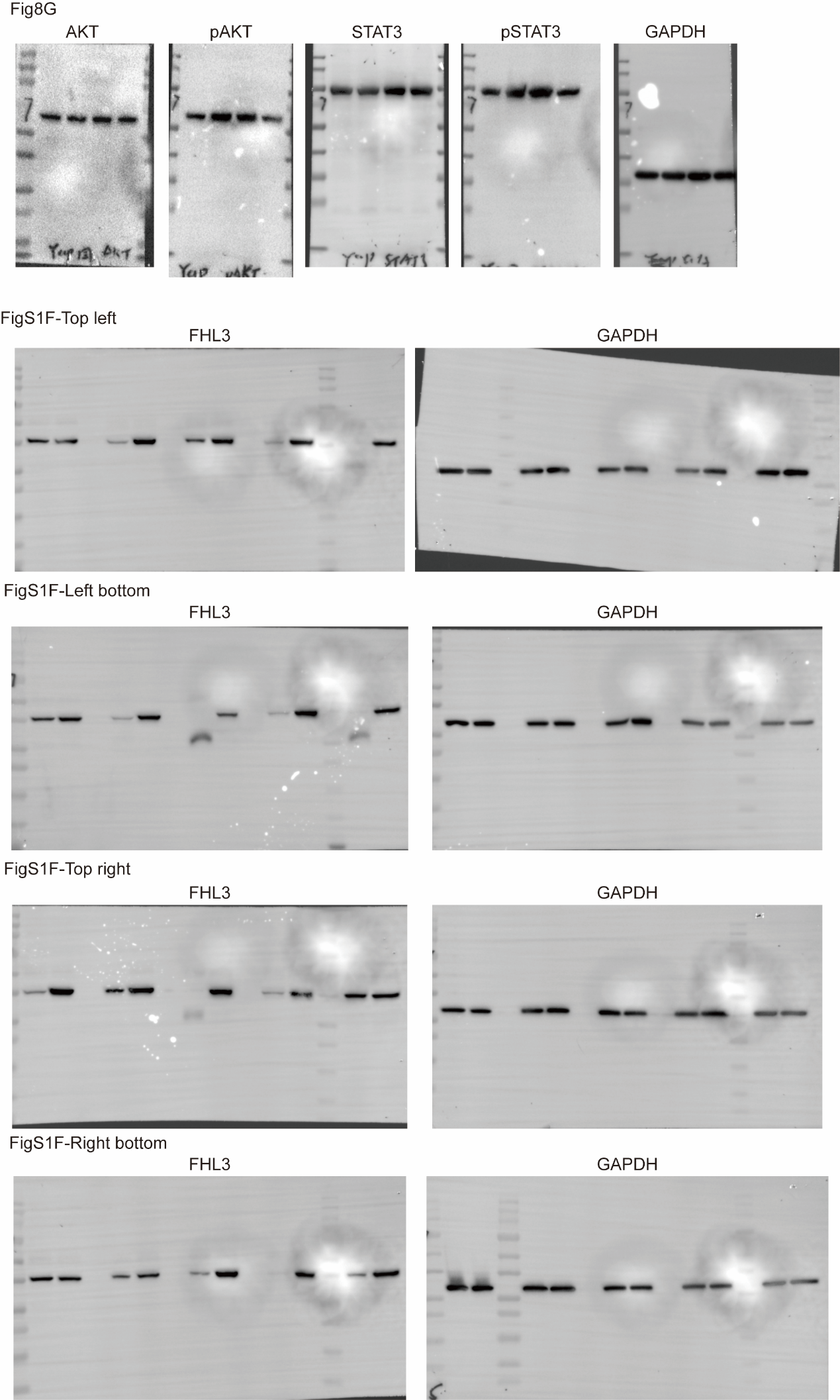

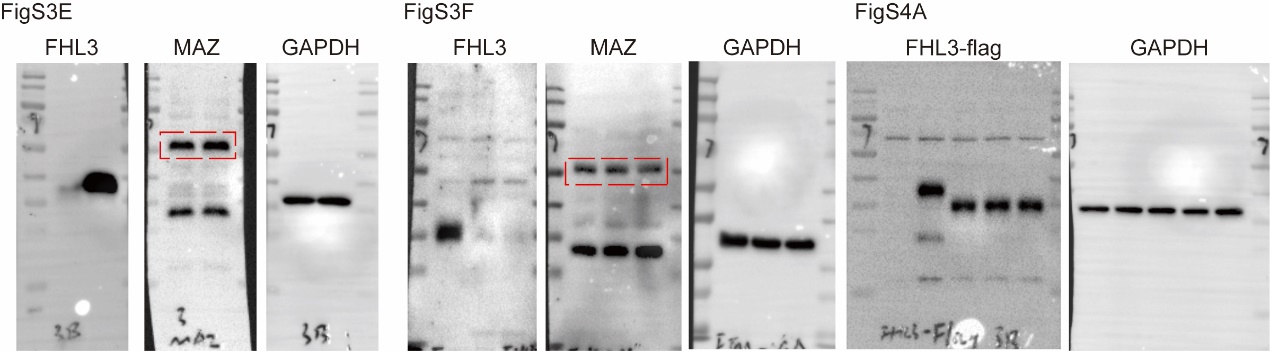

Supplement: Supplementary file 2 — Original western blots [file 41419_2025_8117_MOESM2_ESM.docx]
